# Divergent B-cell repertoire remodelling by mRNA, DNA and live attenuated vaccines in fish

**DOI:** 10.1038/s41541-025-01232-8

**Published:** 2025-07-24

**Authors:** Dean Porter, Catherine Collins, Andrea Mazzolini, Luc Jouneau, Vanessa Mhanna, Céline Coiffier, Mathilde Peruzzi, Yan Jaszczyszyn, Encarnita Mariotti-Ferrandiz, Francois Huetz, Bertrand Collet, Thierry Mora, Aleksandra M. Walczak, Bernard Verrier, Pierre Boudinot

**Affiliations:** 1 Université Paris-Saclay, INRAE, UVSQ, VIM, Jouy-en-Josas, France; 2 School of Biological, Earth and Environmental Sciences, University College Cork, Cork, Ireland; 3 Environmental Research Institute, University College Cork, Cork, Ireland; 4 Laboratoire de physique de l’école normale supérieure (PSL University), CNRS, Sorbonne Université and Université Paris Cité, Paris, France; 5 Université de Paris-Saclay, INRAE, BREED, Jouy-en-Josas, France; 6 Sorbonne Université, INSERM, Immunology-Immunopathology-Immunotherapy (i3), Paris, France; 7 UMR 5305: Laboratoire de Biologie Tissulaire et d’Ingénierie Thérapeutique, Lyon, France; 8 Université Paris-Saclay, CEA, CNRS, Institute for Integrative Biology of the Cell (I2BC), Gif-sur-Yvette, Cedex France; 9 Institut Pasteur, Université Paris Cité, INSERM UMR1222, Antibodies in Therapy and Pathology, Paris, France; 10 Adjuvatis, Batiment Laennec 60, Lyon, France

**Keywords:** Vaccines, Clonal selection

## Abstract

Vaccination is critical for the future of aquaculture, and nucleic acid vaccines have a major potential for fighting emerging fish infectious diseases, yet their mechanisms remain poorly understood. We compared B-cell responses induced by an mRNA, a DNA, and an attenuated vaccine, all encoding the same antigen against a fish rhabdovirus. Rainbow trout IgHμ repertoires were examined to investigate how vaccines reshape clonal composition and complexity of the B-cell repertoire. The attenuated virus drove protection through a small number of highly shared public clonotypes encoding neutralizing antibodies. The mRNA vaccine profoundly remodelled the repertoire in some individuals and induces low, but still protective, neutralising Ab titers without public expansions. The DNA vaccine induced high neutralizing Ab titers, providing full protection with minimal impact on B-cell repertoire. Clustering analysis revealed partial sharing of private responses between fish. These findings highlight profound divergences between fish B-cell responses to nucleic acid and attenuated vaccines whilst all of three vaccines induce protective responses.

## Introduction

The immune system of jawed vertebrates relies on adaptive immune responses driven by antigen (Ag) specific clonal expansions of T and B lymphocytes. Each lymphocyte expresses a single Ag-specific receptor generated by somatic V(D)J gene rearrangement during differentiation. Upon infection, B and T lymphocytes carrying receptors specific to the pathogen’s Ag become activated, proliferate, and differentiate into effector cells, including antibody-producing plasma cells for B lymphocytes^1^. After resolution, persistence of long lived and memory B and T cells specific for the pathogen sustains a long-term immunity that constitutes the basis of vaccination.

The fundamental features of this adaptive immune system emerged in the common ancestor of jawed vertebrates ~450 million years ago and have remained remarkably conserved across all branches of cartilaginous fish and bony vertebrates, including teleost fish and tetrapods^2,3^. However, the organisation of lymphoid tissues and the biology of lymphocytes are very different between fish and mammals. Unlike mammals, fish lack lymph nodes and their B lymphocytes differentiate in the pro- and meso-nephros rather than in the bone marrow^4,5^. In contrast to the classical dogma stating the absence of germinal centers (GC) in fish, recent studies have identified the existence of inducible teleost melanomacrophage centers-associated lymphoid aggregates (M-LAs) with features analogous to endotherm GCs^6,7^. These structures, observed within fish spleen and pronephros, support the induction of systemic antibody (Ab) responses and somatic hypermutation of immunoglobulin (Ig) genes involving both B and T cells. The long-term survival niche for fish plasma cells is thought to be the pronephros^8,9^. In the absence of lymph nodes, how Ag is captured by antigen presenting cells and presented to B and T cells remains poorly understood^10^, as is the biology of fish dendritic cells. Systemic Ab responses in teleost species are primarily mediated by IgM, with two additional Ig classes: IgD, whose function remains elusive^11^ and IgT, a fish specific immunoglobulin specialised in mucosal immunity^12^. Similar to mammals, fish exhibit a faster and stronger secondary response (i.e., higher Ab titers) after repeated immunisations^13–15^, opening up the possibility of vaccinations to protect against infection. Importantly, fish are ectotherms as such their immune response is strongly affected by the temperature of the environment, especially for T cells^16^. The implication of the profound anatomical, physiological, and genetic divergences between the immune systems of fish and mammals is still largely unknown. Therefore, analysing fish responses to various vaccines is important, both for practical applications and from an evolutionary perspective.

Vaccination plays a pivotal role in combatting the spread of pathogens in aquaculture, minimising their harmful effects on the health of farmed fish species. Multiple vaccine platforms have been developed for use in fish comprising inactivated, attenuated, subunit, and nucleic acid vaccines^17,18^. Inactivated vaccines were first developed and widely used to protect Atlantic salmon against bacterial diseases^18^. Attenuated vaccines efficiently protect against several viral diseases and generally induce very good protection but are associated to safety issues. DNA vaccines have proved to be particularly effective in protecting against Rhabdoviruses such as the viral haemorrhagic septicaemia virus (VHSV) experimentally^19,20^ and two commercial DNA vaccines are available for use in aquaculture^17^. mRNA vaccines for fish are still in their infancy^21^ but we have recently reported a protective mRNA vaccine formulated in liponanoparticles (LNPs) against VHSV^22^. Despite their widespread use in aquaculture, the adaptive immune mechanisms underlying vaccine-induced protection, as well as the molecular and cellular basis of immune responses, remain poorly understood.

To get insights into vaccine-induced immune memory and protection, we first undertook the characterisation of the B cell response induced by a protective attenuated VHSV vaccine^23^ through repertoire sequencing^24^. We identified a strong public IgHμ clonotypic response directed against the VHSV in a clonal line of rainbow trout (*Oncorhynchus mykiss*). These public anti-VHSV clonotypes pre-existed at low frequency before infection^25^, and were prominently expanded during the response in the spleen^24,25^ and pronephros^26^. Importantly, they encode the heavy chain of VHSV neutralising Abs^27^, which are critical for protection, as neutralising Abs are typically associated with host protection during rhabdovirus infections^19,28,29^.

In this study, we aimed to compare B cell responses induced by this attenuated live vaccine and by nucleic acid vaccines. Direct studies into the differences in clonal expansion between different vaccine platforms are limited even in mammalian models. Comparisons of B cell repertoires after multiple vaccinations with mRNA vaccines against COVID-19 show that multiple doses promote the expansion of receptor-binding domain-specific memory B cells with an increased affinity to the binding domain of clonotypes that were persistent between vaccinations^30^. The level of titers of these specific antibodies has also been shown to be relatively low, demonstrating the effectiveness of rare but reoccurring antibodies specific to the receptor-binding domain^31^. Whilst recent studies have shown that vaccination with the mRNA vaccine in comparison to the COVID-19 virus itself drives a narrower clonotype expansion^32,33^. However, the differences in response between DNA and mRNA vaccines, if any, are yet to be clearly defined. To address this, we leveraged protective DNA and mRNA vaccines against VHSV in rainbow trout, with both vaccines encoding the exact same glycoprotein Ag as the attenuated vaccine used in our previous studies. Using a rainbow trout doubled haploid isogenic line, ensuring optimal comparability of IgHμ repertoires across genetically identical individuals, we analysed the clonal composition and dynamics of the IgM+ B cell response to these three vaccines. Our findings reveal that protection induced by mRNA, DNA and attenuated vaccines is associated with different levels of Abs responses, and that each vaccine exerts a unique impact on the spleen IgM repertoire. While mRNA vaccine leads to the highest repertoire perturbation, large public expansions encoding neutralising Abs are found only after vaccination with the attenuated virus and are marginally expressed in response to nucleic acid vaccines. These insights deepen our understanding of nucleic acid vaccine mechanisms and provide a framework for comparing immune responses across fish and mammals.

## Results

### Nucleic acid vaccines’ protection against VHSV is driven by specific IgM antibodies in serum

The production of antibodies by the host against VHSV is vital for protection. We first carried out serum neutralisation and ELISA assays on the serum of fish 3-months post prime immunisation using DNAgVHSV^19^, LNP (RNAgVHSV)^17^, or live attenuated VHSV vaccine (AttVHSV)^23^. At day 90, serum from all three vaccines induced neutralisation against VHSV. Fish vaccinated with either DNAgVHSV or LNP (RNAgVHSV) showed a partial reduction of plaques, whereas the attenuated vaccine led to an almost complete reduction compared to PBS injected controls (Fig. S1A). Fish vaccinated with either the DNAeGFP or LNP (RNAeGFP) showed no reduction in plaques. Importantly, adding the anti-trout IgM monoclonal Ab 1.14^34^ abolished the neutralisation, confirming that it was due to the presence of anti-VHSV IgM Abs and not to other components of the serum, as previously reported in (Castro et al., 2013, 2021) (Fig. S1A). Interestingly, the levels of circulating anti-VHSV antibodies (Abs) in the serum were not increased compared to PBS injected fish in both the DNAgVHSV and LNP (RNAgVHSV) vaccinated groups (Fig. S1B). In contrast, these levels were significantly higher in the group vaccinated with the attenuated vaccine.

### Similar IGHV and J gene usage in the spleen IgH repertoire across different vaccine groups

**Fig. 1:**
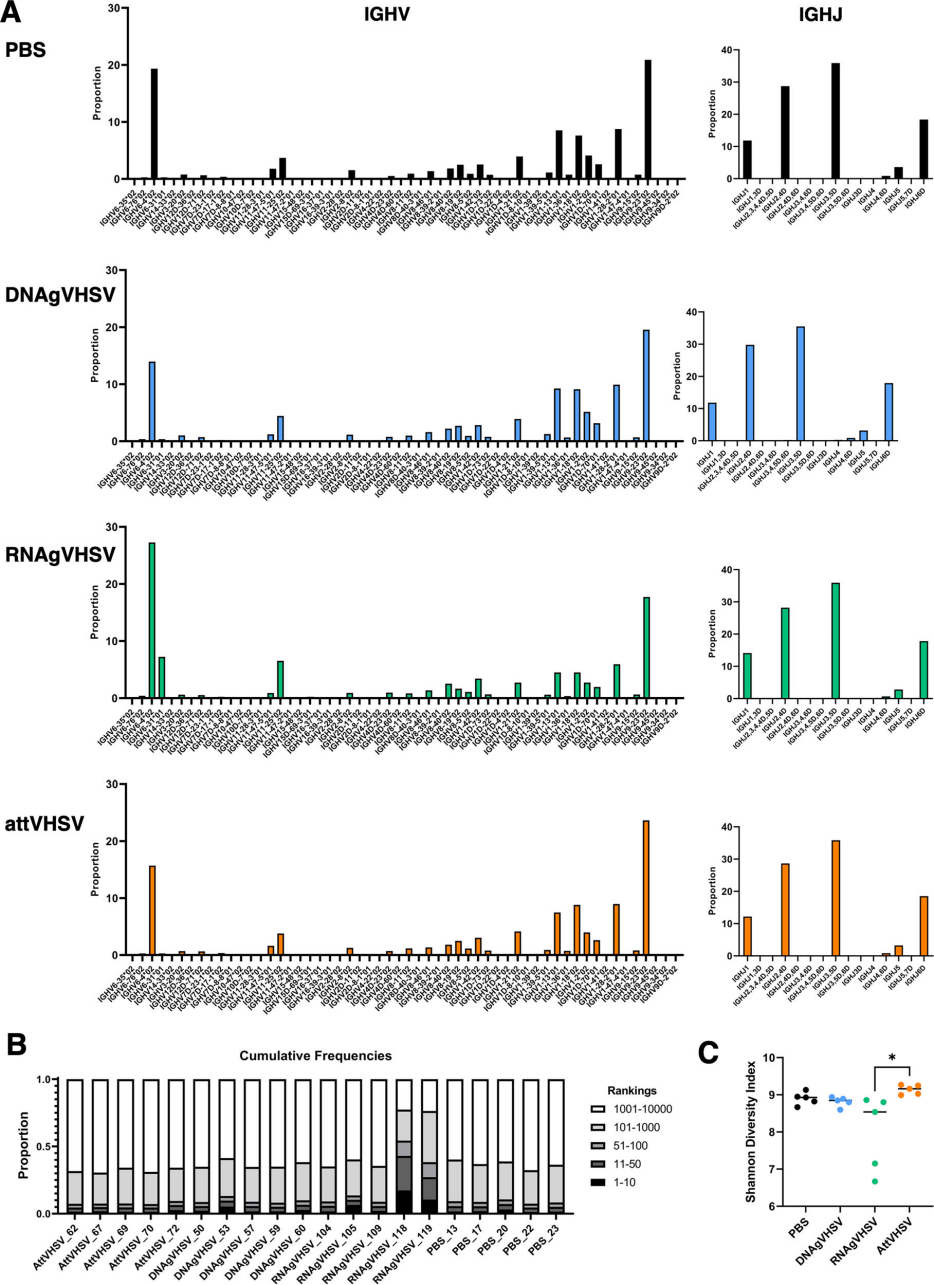
Characteristics of the clonotype composition three months after vaccination with nucleic acid vaccines. **A** IGHV and J gene usage for each vaccine type. **B** Frequency distribution of IgHμ clonotypes, categorised into rank ranges, is shown for each fish. The repertoire corresponding to the top 10 most frequent clonotypes is shown in black. **C** Measures of clonotype diversity between different vaccination types using the Shannon Diversity Index.

The diversity of B cell repertoires is shaped by IGHV/J gene usage and IGH CDR3, which vary due to different probabilities of rearrangement, IgH/IgL pairing constraints and B cell selection processes. We examined the repertoires of IgM B cell from the spleen between all three vaccinated groups in comparison with the PBS injected fish (Fig. 1, Fig. S2). Figure 1A shows IGHV/J gene usage three months post vaccination. Largely the IGHV usage is very similar amongst all groups with no major differences other than IGHV6-31 in LNP (RNAgVHSV) vaccinated fish. Principle component analysis illustrates PBS, DNAgVHSV, and AttVHSV groups share comparable profiles, whereas the RNAgVHSV group shows a relatively distinct pattern with higher variance (Fig. S2B).

No clear differences in IGHJ gene usage and CDR3 length (Fig. S2A) were observed. The proportion of the expressed repertoire attributed to the most frequent clonotypes (Fig. 1B, Top100 clonotypes) revealed generally similar frequency distributions after PBS injection and after vaccination with AttVHSV, DNAgVHSV, or RNAgVHSV; however, two of five LNP (RNAgVHSV) vaccinated fish (RNAgVHSV_118 and _119) displayed marked expansions, with their top 100 clonotypes comprising 40–50% of the repertoire. These outliers also showed reduced diversity indices (Figs. 1C, S2C), skewing the group’s overall diversity metrics. Overall, vaccination generally did not strongly modify the global structure of the spleen expressed IgHμ repertoire, as previously reported by Castro et al. at 5 months post vaccination with the attenuated vaccine^26^, but exacerbated responses to the LNP (RNAgVHSV) vaccine may occur.

### Clonotype sharing and expression across vaccine groups

Expanded responsive clonotypes are expected to be shared by fish within groups (convergent/public responses), or to be among the most frequent clonotypes in each repertoire of individual vaccinated fish (private responses).

**Fig. 2:**
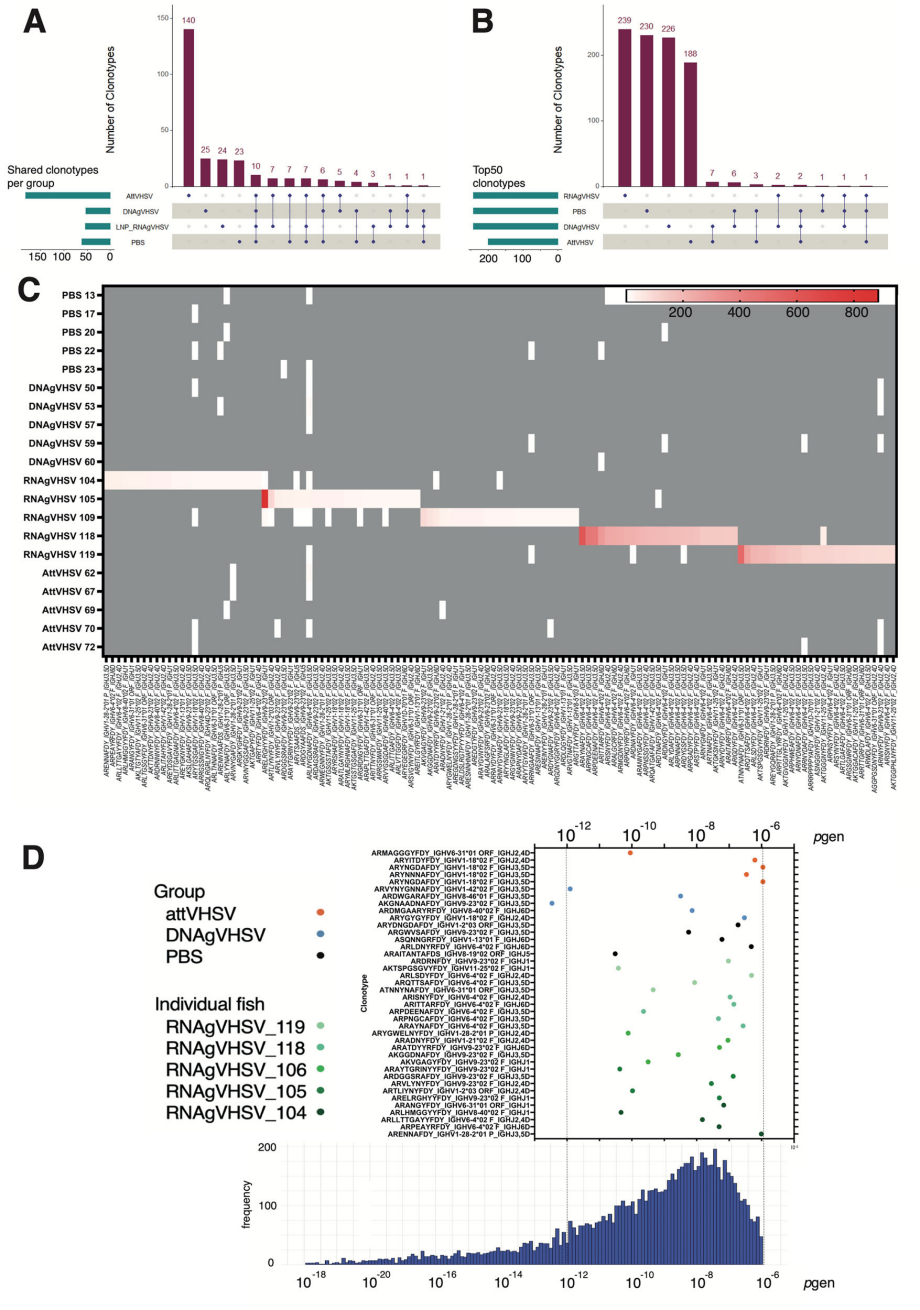
Distinct private responses of the LNP (RNAgVHSV) vaccine. **A** UpSet plot illustrating highly shared clonotypes, defined as those detected in four or five fish per group based on MID counts. The left panel shows the number of clonotypes meeting this shared criterion within each vaccination group. The right panel presents the number of clonotypes shared between groups (top) and the selected pairwise comparisons (bottom). **B** UpSet plot showing the top 50 most abundantly expressed clonotypes for each vaccination group. The left panel shows the number of clonotypes in the top clonotype lists within each vaccination group. The right panel presents the number of clonotypes shared between groups (top) and the selected pairwise comparisons (bottom). Both UpSet plots are based on data from a subsampling of 100,000 sequences per condition. **C** The top 25 clonotypes identified in each RNAgVHSV-vaccinated fish, highlighting the unique private immune responses elicited by the vaccine. The heat map colouring represents the MID count, with red indicating 800 or more reads and grey indicating the absence of a clonotype. **D** Probability of generation of the top 5 clonotypes from each RNA vaccinated fish compared to the top 5 clonotypes across all 5 fish in the DNA, PBS and attVHSV vaccinated groups (upper panel). The lower panel shows *pgen* distribution of clonotypes from PBS samples. * denotes significant difference from the PBS distribution where *p* < 0.01 by a one or two-sided Kolmogorov–Smirnov test.

Figure 2A illustrates the degree of clonotype sharing among different vaccine groups, in comparison to the control group (PBS) (horizontal green bars). To identify highly shared clonotypes between fish, sharing was defined as a molecular identifier (MID) count of ≥1 in 4 or 5 fish per vaccination group, based on a subsampling of 100,000 MID counts per fish. Notably, AttVHSV-vaccinated fish exhibited the highest degree of clonotype sharing, with 183 clonotypes shared across four or more fish (Fig. 2A, horizontal green bars). In contrast, the DNAgVHSV and LNP (RNAgVHSV) vaccinated fish demonstrated much lower levels of sharing between fish, 53 and 54 clonotypes respectively, similar to the level sharing seen within the PBS control group, 61 clonotypes (Fig. 2A, horizontal green bars). This finding indicates that AttVHSV likely induces a more convergent immune response across individuals, with a greater importance of public components. When comparing clonotype sharing patterns across different vaccine groups (vertical purple bars), the majority of group-shared clonotypes were found to be unique to each specific vaccination type. Some evidence of sharing was observed between groups immunised with different vaccines. However, 38 clonotypes were shared between vaccinated and control fish, while only 14 were shared between vaccinated groups (and not by controls), with only one clonotype being shared by all three vaccinated groups. Shared clonotypes tended to primarily involve the *IGHV1-18*, *IGHV6-4*, and *IGHV6-31* gene families. While overall responses are vaccine-specific, certain *IGHV* genes/subgroups are therefore preferentially involved in shared expansions across different vaccine types, including *IGHV1-18* which is expressed in known anti-VHSV public clonotypes^25,27^.

The distribution of the most frequent clonotypes was then investigated across individual fish within and between experimental groups (Fig. 2B). To do so, the 5 “Top50” most expressed clonotype sets from each of the 5 fish in each group were aggregated to obtain a non-redundant list (TCL for Top Clonotype Lists). The total number of clonotypes in a TCL was thus between 50 (if all were shared by the 5 fish of the group), and 250 (if none were shared). Figure 2B (horizontal bars) shows that TCL for PBS-ctrl, DNAgVHSV, and LNP (RNAgVHSV) comprised almost 250 clonotypes, indicating that virtually all of the Top50 clonotypes were unique to individual fish. Figure 2C illustrates that even the top 25 most frequent clonotypes of the two fish with very strong responses induced by LNP (RNAgVHSV) (fish #118 and #119) were poorly shared by other vaccinated fish, not significantly more than with controls. Whilst for the AttVHSV-vaccinated fish, the TCL (*N* = 188) revealed clonotype sharing evoking the known public response (Fig. 2B). The sharing of TCL clonotypes between groups was also minimal (Fig. 2B, vertical bars), again indicating that each vaccine generates a distinct top clonotypic signature, reflecting unique modulation of the B cell repertoire. The limited sharing of dominant clonotypes highlights vaccine composition on shaping specific and diverse B cell responses. The public response dominant in fish vaccinated by the AttVHSV vaccine^24–26^ was apparently not prominent within the most expressed clonotypes after immunisation with nucleic acid vaccines.

VDJ recombination is a highly variable process, resulting in a broad range of generation probabilities for different clonotypes. The probability of generation (*pgen*) quantifies the likelihood of a specific VDJ rearrangement being produced by the recombination machinery (see Methods for the accurate definition of *pgen* of an AA clonotype). Figure 2D displays the *pgen* values for the top 5 expressed clonotypes in each fish in the LNP (RNAgVHSV) group, demonstrating that the majority of these highly expanded clonotypes do not correlate with high *pgen* values. Interestingly, the top frequent clonotypes of DNAgVHSV vaccinated fish follow a similar distribution. In general, the data from nucleic acid vaccinated fish shows that the *pgen* of top expanded clonotypes are not particularly large, which is consistent with a random draw. However in 2 of the LNP (RNAg:gVHSV) fish, 104 and 118, we saw evidence of two highly expanded clonotypes (one in each fish) with high *pgen* values, ARENNAFDY_IGHV1-28-2*01 P_IGHJ3,5D and ARAYNAFDY_IGHV6-4*02 F_IGHJ3,5D respectively. Strikingly, the highly expanded clonotype in fish #119 was not associated with a high *pgen*, showing it is not always the case. We also showed previously that *pgen* of nucleotide sequences coding for the eight public amino acid clonotypes dominant in the response induced by the attenuated vaccine were at the top of the distribution of *pgen* of VH1-18(V)J rearrangements^25^. We observed three of the amino acid public clonotypes found within the top 5 of the attenuated VHSV fish (Fig. 2D). Among all tested groups, only the attenuated VHSV vaccine group exhibited a statistically significant difference in the *pgen* distribution compared to PBS, based on the two-sided Kolmogorov–Smirnov test (*p* = 0.0077) (Fig. 2D). A one-sided test further confirmed that the *pgen* distribution in the attenuated group was significantly greater than that of PBS (*p* = 0.0039). No significant differences were observed between the DNA or RNA vaccinated groups and PBS, regardless of whether a two-sided or one-sided test was used. Together, these results demonstrate that nucleic acid-based vaccines and the live attenuated virus vaccine induce Ab responses with different importance of public/shared and private components.

### DNAgVHSV and LNP (RNAgVHSV) vaccines elicit expansion of the known public anti-VHSV clonotypes in most individuals, but at much lower frequencies than the attenuated vaccine

**Fig. 3:**
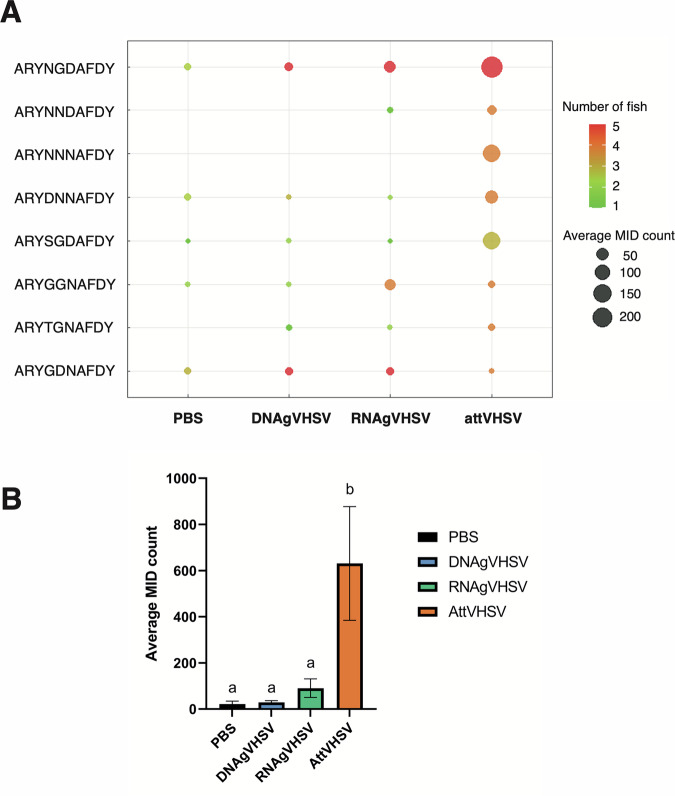
Public clonotypes against VHSV. **A** Bubble plot displaying the public response to VHSV in each vaccination group. Bubble size corresponds to the average frequency of MID counts across fish in each vaccination group, while bubble colour indicates the number of fish expressing each clonotype. All 8 public clonotypes belong to the IGHV1-18*01 and IGHJ3 families with their respective CDR3 regions being shown. **B** Bar chart showing the average sum of MID counts relating to all 8 public clonotypes. ^a^ and ^b^ denote significant differences after one-way ANOVA where *p* < 0.05. Data are based on a subsampling of 100,000 MID counts per fish.

A strong public anti-VHSV response has been previously identified in spleen and head kidney of fish immunised with the attenuated VHSV vaccine; this response involves eight IGHV1-18*02/IGHJ3 IgHμ clonotypes containing highly similar CDR3 sequences^24–26^. In this study, we sought to identify if these same public components were expressed after nucleic acid vaccination (Fig. 3A). As previously observed, the group vaccinated with attenuated VHSV showed high levels of expression of all 8 clonotypes, of which nearly all were shared by over 80% of fish, with IGHV1-18*02_IGHJ3_ARYNGDAFDY being the most abundant. Both the LNP (RNAgVHSV) and DNAgVHSV vaccinated fish also expressed anti VHSV-public clonotypes in most fish, but at significantly lower levels than fish vaccinated with the attenuated virus (Fig. 3B). Strikingly, one of the public clonotypes (IGHV1-18*02_IGHJ3_ARYGDNAFDY) was expressed in both the DNAgVHSV and LNP (RNAgVHSV) vaccinated fish at higher counts than in fish vaccinated with the attenuated virus, and IGHV1-18*02_IGHJ3_ARYGGNAFDY clonotype was also found at substantial frequencies in the LNP (RNAgVHSV) group. These anti-VHSV public clonotypes were found at low level in a number of controls, in line with their high *pgen*. These public clonotypes in PBS injected fish tended to be detected at low “pre-expansion” frequencies as previously observed^24–26^. However, the average frequency of public clonotypes was not significantly different between controls, DNAgVHSV and LNP (RNAgVHSV) vaccinated fish (Fig. 3B). A low frequency IGHV1-18*02_IGHJ3 public response to DNA vaccines was also observed in an independent experiment testing the DNAgVHSV vaccine alone (average frequency of 2.5 E-4) compared a typical frequency of 0.001 to a few percent in attenuated vaccinated fish in the present experiment (Supplementary Text).

### Patterns of clonotype sharing in responses to the three vaccines confirm the lack of strong public component in the response to nucleic acid vaccines

**Fig. 4:**
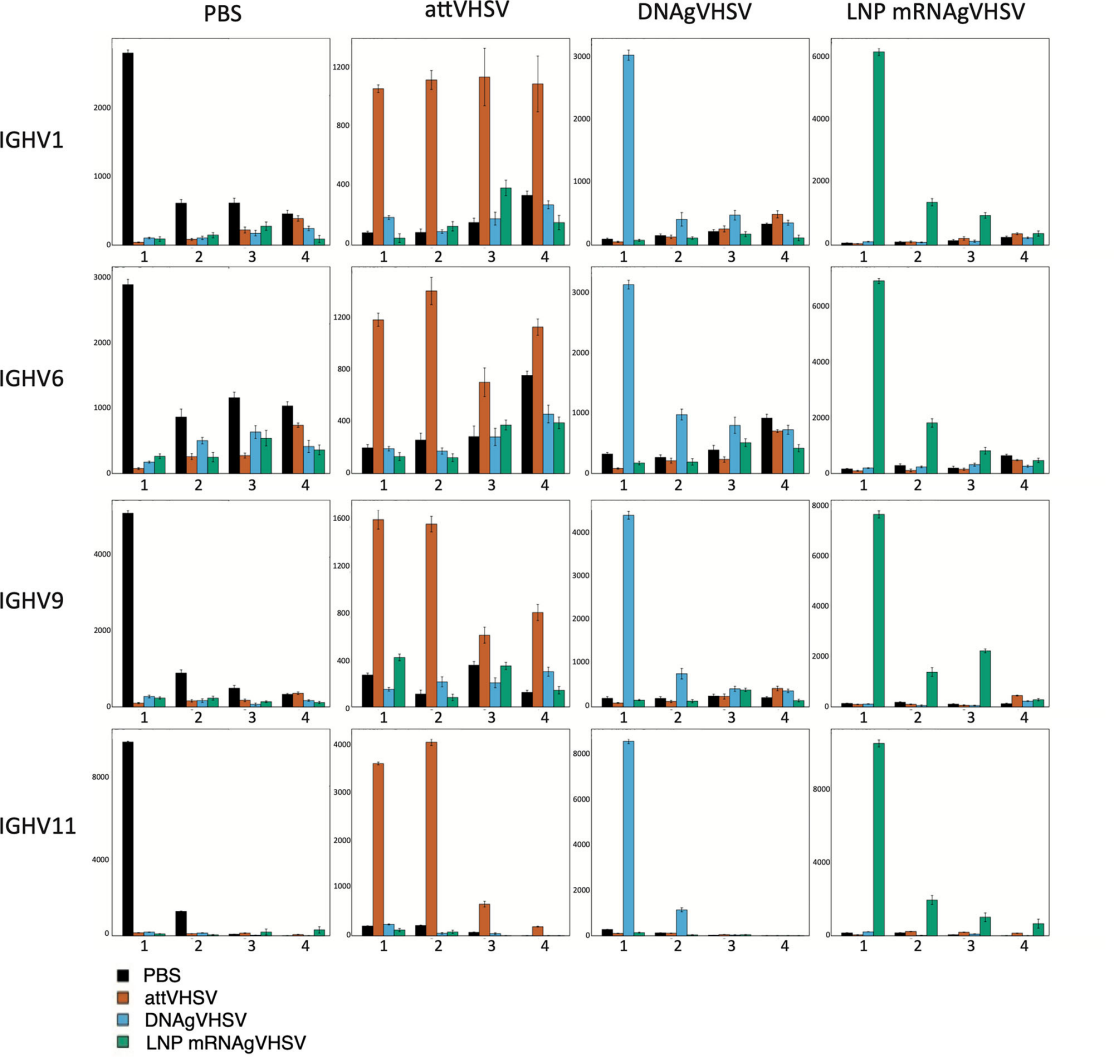
Highly shared clonotypes within IGHV subgroups. Barplots showing cumulative expression of the top 50 clonotypes shared by n individuals (i.e., present in 1, in 2, in 3 or in 4 fish), for a given IGHV subgroup, within each vaccinated group after three months post initial vaccination. Individual barplots show total expression and sharing of the TCL clonotypes for that condition compared to other conditions with PBS (in black), AttVHSV (in orange), DNAgVHSV (in blue) and RNAgVHSV (in green). X axis (1, 2, 3 and 4) demonstrates the level of sharing within each condition. Y axis represents cumulative expression based on the average MID count of 10 subsamplings of 10,000 per individual. To improve visualization of sharing at the VH subgroup level, we excluded the fish with the lowest MID counts for certain VH subgroups in each vaccination group from this part of the analysis, this was also done at the VH gene level. See Fig. S3 for IGHV subgroups where subsampling was possible.

To further investigate the composition of the IgM+ B cell response induced by vaccination at higher resolution, we compared the contribution of shared/public clonotypes to the repertoire for each VH subgroup and each fish, three months post vaccination. Focusing on non-redundant lists of Top50 most frequent clonotypes for each VH subgroup and each experimental group, (TCL as defined above), we analysed the presence of clonotypes across fish of each experimental group, or across fish of different groups. The sharing level between different fish was then shown in a bar plot as the *cumulated frequency* of Top50 IgHμ clonotypes present in *n* individuals (*n* = 1, 2, 3, 4) within a given group (i.e. in 1, 2, 3 or 4 individual fish) (Fig. 4). TCL clonotypes from controls are represented as dark blue bars, those from AttVHSV in orange bars, those from DNAgVHSV in cyan bars and those from LNP (RNAgVHSV) in green bars (Fig. 4). These plots therefore combine sharing and expression level to provide an overview of the contribution of the TCL clonotypes of a group to the IgHμ repertoire in different conditions.

**Fig. 5:**
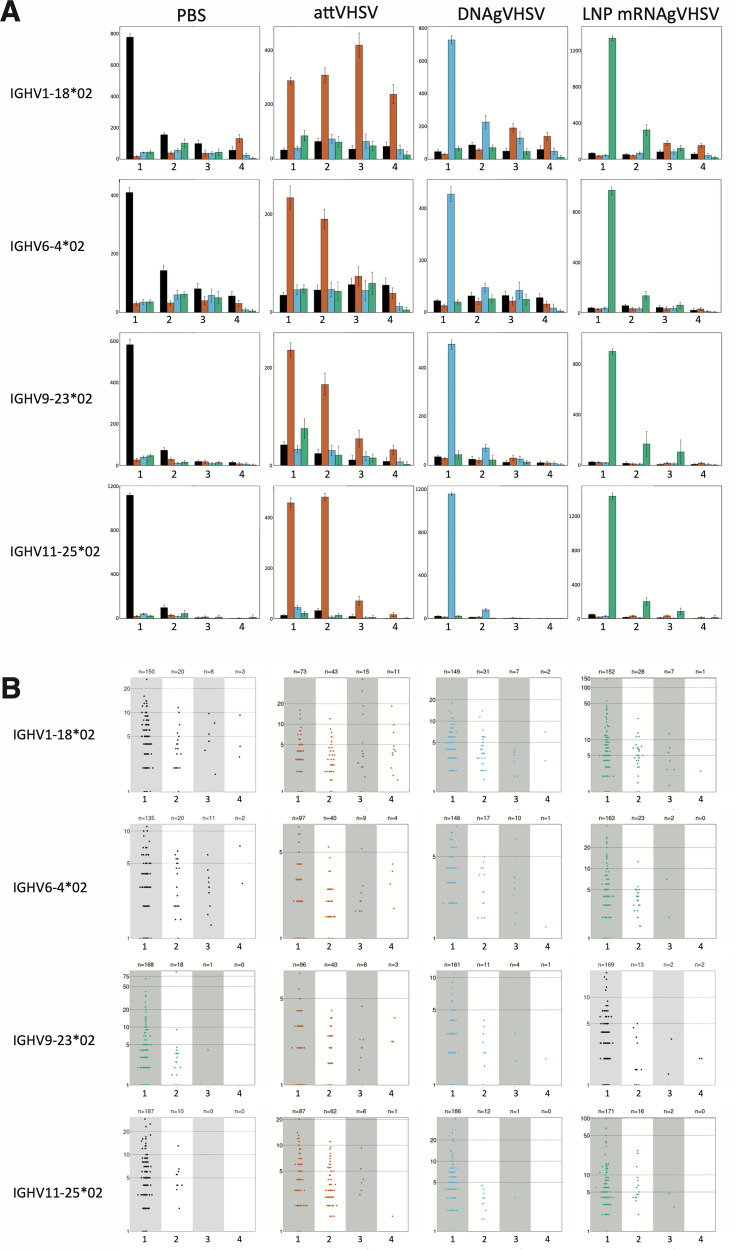
Highly shared clonotypes at the IGHV gene level. **A** Bar plots showing cumulative expression of the top 50 clonotypes shared by n individuals (i.e., present in 1, in 2, in 3 or in 4 fish), for a given IGHV *gene*, within each vaccinated group after three months post initial vaccination. Individual bar plots show total expression and sharing of the TCL clonotypes for that condition compared to other conditions with PBS (in black), AttVHSV (in orange), DNAgVHSV (in blue) and RNAgVHSV (in green). X axis (1, 2, 3 and 4) demonstrates the level of sharing within each condition. Cumulative expression is based on the average MID count of 10 subsamplings of 1000. **B** Dotplot showing the distribution of clonotype frequency at different levels of sharing (n = in 1, in 2, in 3 or in 4 fish) between the top 50 clonotypes for each vaccinated group with the average MID count also shown. The data are calculated based on the average MID count of 10 subsamplings of 1000 per individual. See Fig. S4 for all IGHV gene bar plots where subsampling was possible.

The comparison of sharing patterns for each VH subgroups points to the main contribution of public clonotypes to the VH1 repertoire after AttVHSV vaccination (these clonotypes were absent in controls), and also uncovers lower contributions of public components to VH9 and VH11 responses to this vaccine (Fig. 4). To analyse clonotype sharing at a higher resolution, the same analysis performed at the VH *gene* level showed that all VH1 public clonotypes express VH1-18, and one of the public CDR3 previously identified. As seen above in the targeted analysis this public response has a plain but modest contribution to the IgHμ repertoire of DNA and mRNA vaccinated fish (Fig. 5A, top line, orange bars in 3rd and 4th bar plots). Respective contributions of the top shared clonotypes of the response to the attenuated vaccine is illustrated on Fig. 5B, top line, second column. Notably, Top50 VH1 clonotypes from DNA (resp. mRNA) did not contribute to the repertoires of other vaccinated groups. While the lack of public response for all the other VH subgroups had been observed previously after AttVHSV vaccination, we show here that DNA and mRNA vaccines do not lead to strong public response within any VH subgroup. The pattern observed for VH6 (Fig. 4), with clonotypes highly shared after AttVHSV vaccination being also present and well expressed in all/most fish in controls and found again after DNA vaccination, is reminiscent of our previous observations^26^. However, these clonotypes were not shared after mRNA vaccination suggesting a different perturbation of the repertoire in this experimental group.

While no significant expression of public clonotypes expressing a VH9 gene could be detected after AttVHSV or DNA vaccination, a response shared by 3 fish was observed after mRNA vaccination (Fig. 4). Several clonotypes relating to IGHV9-23*02 F were found shared between 3 or more fish, commonly these clonotypes were found relatively low expressed (<100 MID counts per fish) however one clonotype ARELRGHYYFDY_IGHV9-23*02 F_IGHJ1 was found to be largely expanded (>4000 MID count) in one fish (LNP (RNAgVHSV)_105). Another interesting observation is that clonotypes belonging to the VH11 TCL after AttVHSV vaccination were expressed by 4 or more fish after mRNA vaccination (Fig. 4, IGHV11 line, 2^nd^ and 4^th^ panels). These clonotypes related to the IGHV11-25*02 gene and were found at modest levels of expression (<50 MID counts per fish). Additionally, the increase of the cumulated expression scale after mRNA vaccination in the VH1, VH9 and VH11 (Fig. 4) subgroups was compatible with a strong perturbation/response in some fish at least, in line with Fig. 1B, C.

**Fig. 6:**
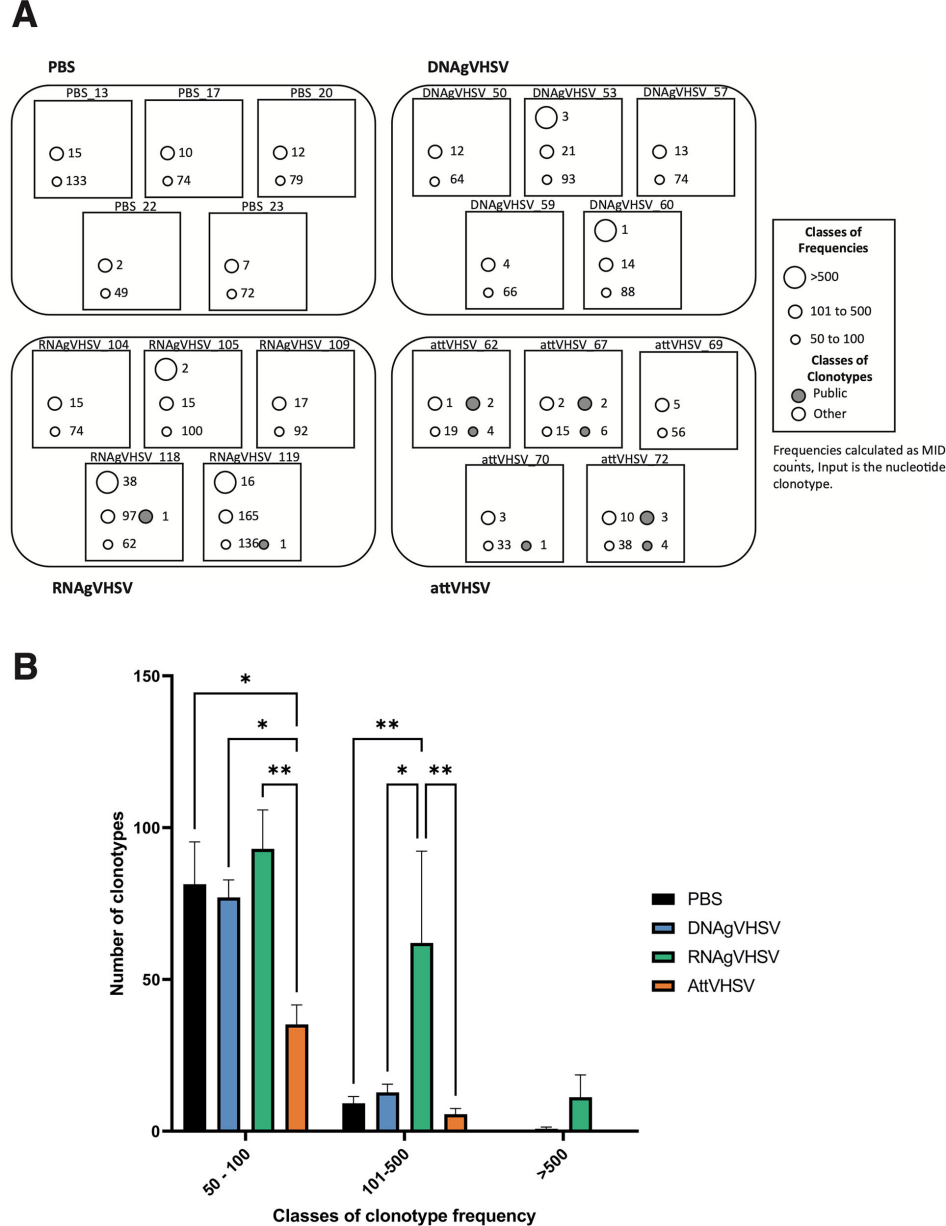
Classes of clonotype frequencies. **A** Schematic representation of the top expressed private clonotypes (empty circles), and public VH1-18-JH3 clonotypes (grey circles). Circle size indicates the frequency class of a given clonotype, with the number next to each circle representing the number of clonotypes within that class. **B** Bar plot showing the distribution of clonotype frequency classes (both public and private). Frequencies are based on MID counts, summing both public and other responses. Clonotype classification is derived from nucleotide sequence data. Frequency classes are arbitrarily defined for visualization purposes. Data are based on a subsampling of 20,000 MID counts per fish.

We also analysed the frequencies of the most frequent IgHμ clonotypes in each fish of each experimental group (Fig. 6A). Large expansions (found > 100 MID counts in a subsampling of 20,000) were mostly found after mRNA and, to a lesser extent, DNA vaccination. In contrast, frequent clonotypes were less frequent in controls, and curiously, even more so after AttVHSV (at this time point, three months post-injection) (Fig. 6B) suggesting completely different dynamics of clonal expansion between the different vaccinations. The intensity of the *IGHV1.18 – IGHJ3* public response followed a completely different pattern, being found mainly after vaccination with the attenuated virus.

### Clustering clonotypes into convergent clusters identify private responses shared by several mRNA vaccinated fish

**Fig. 7:**
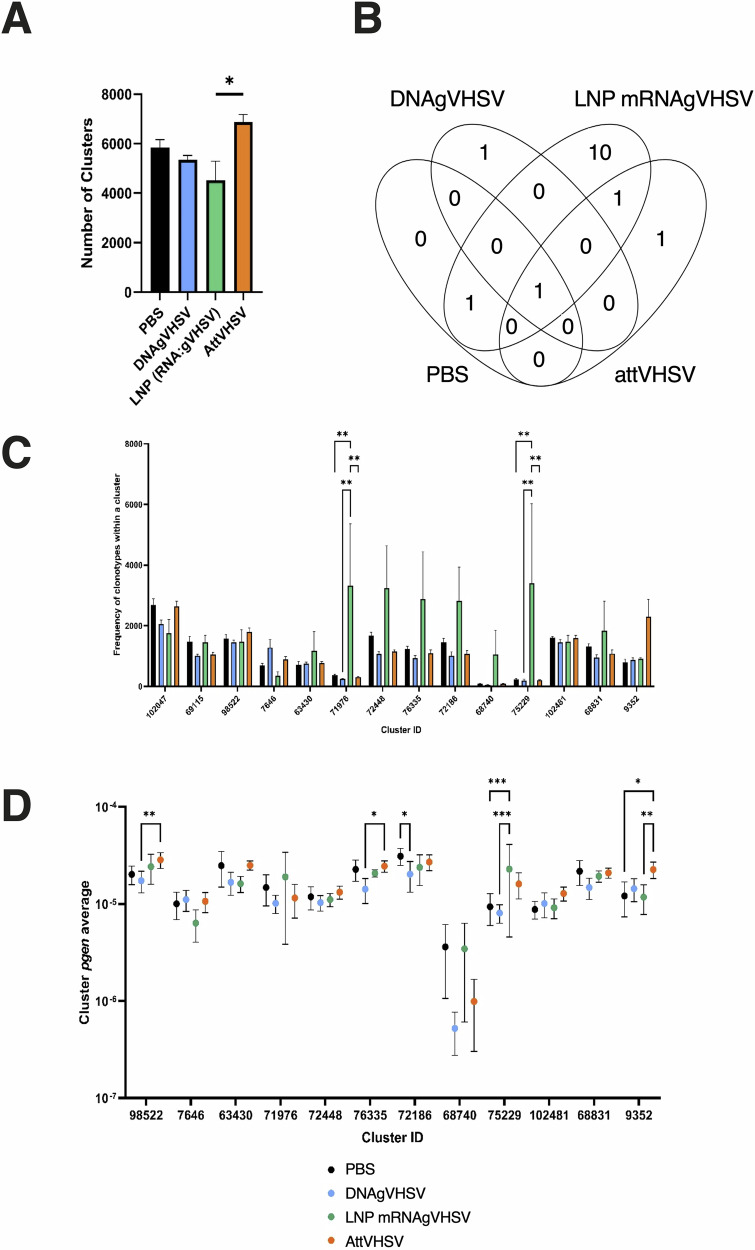
Clonal clustering analysis. **A** Bar chart showing average number of clusters found per vaccine condition. **B** Venn diagram showing the top expressed clusters (>2000 MID sum in at least one fish per vaccination group). **C** Bar chart showing average frequency of clonotypes within each cluster of interest per vaccination group. **D** Dot plot showing the average per group of “cluster *pgen*”. We define the cluster *pgen* as the sum of *pgen* associated to all clonotypes detected within each individual for a cluster of interest.

To further assess sequence similarity within the immune repertoire, we used a clustering method, which groups together functionally similar sequences^35^. Clusters were created by comparing the amino acid-sequences similarity of CDR3s, and clonotypes with similar or identical CDR3 sequences but differing IgHJ or IgHV regions being assigned to separate clusters. This approach allowed the detection of potential antigen-specific responses, including those composed of non-public clonotypes. Figure 7A illustrates the number of clusters identified per group, revealing a significant difference between RNAgVHSV- and AttVHSV-vaccinated fish. This difference was primarily driven by a reduction in clusters observed in RNAgVHSV_118 and RNAgVHSV_119, both of which exhibited a remarkable increased expression of the top clonotypes and decreased clonotype diversity. To further investigate the contribution of highly expressed clusters, we compared clusters with a total expression greater than 2000 MID counts per group (Fig. 7B). This analysis identified a few highly expressed clusters present in the PBS injected fish; Cluster 102047 (ARDGGNAFDY_IGHV9_2302_IGHJ3,5D) present in all groups and Cluster 69115 (ARLTGGNVFDY_IGHV6-402F_IGHJ2,4D present in both the PBS and RNAgVHSV-vaccinated fish, indicating their presence in the baseline repertoire. Notably, the RNAgVHSV group exhibited 10 unique clusters (Clusters 63430, 97206, 71976, 72448, 76335, 72186, 68740, 75229, 102481, and 68831), while the DNA and AttVHSV groups each had a single unique cluster (Cluster 7646 and Cluster 9352, respectively). The amino acid sequence logos for these clusters are provided in Fig. S5. To identify dominant clusters within each group, we compiled the top 25 clusters from each individual and combined them into a Top Cluster List (TCLus) per group (Fig. S6). This showed us there was a high number of shared clusters between all groups, but that each vaccination group drove unique responses, particularly the RNA vaccinated fish. The summed expression of the highly expressed clusters was then compared between groups (Fig. 7C), highlighting significantly increased expression of Clusters 71976 (ARLNNAFDY_IGHV6-4*02 F_IGHJ2,4D)* and 75229 *(ARLYNAFDY_IGHV6-4*02 F_IGHJ3,5D) in RNAgVHSV-vaccinated fish. These clusters exhibited highly similar amino acid sequences but differed in IGHJ gene usage. Sequences of rainbow trout IGHJ genes are highly similar, and these clusters therefore likely participated to a convergent response to the mRNA vaccine. While not statistically significant, Cluster 9352 (ARYNGNADFY_IGHV1-18*01 F_IGHJ3,5D) showed increased expression in the AttVHSV group and contained several clonotypes associated with the public response.

To establish whether these highly expressed clusters were highly probable, the sum of *pgen* was calculated for each fish within each group based on the clonotypes present within each cluster. This analysis assessed whether the large private expansions of the LNP (mRNAgVHSV) vaccinated fish or the public response had a significant impact on the total *pgen* values. Cluster 9352, associated with the public response, showed a significant increase in the sum of *pgen* values in the attVHSV vaccinated fish compared to both the LNP (mRNAgVHSV) and PBS groups indicating a large expansion of highly probable clonotypes (Fig. 7D). Attenuated VHSV also led to increased sum of *pgen* values for several other clusters, including clusters 76335 and 98522 compared to DNAgVHSV vaccinated fish and also in cluster 63430 compared to the LNP (mRNAgVHSV) vaccinated fish. It is of note that these three clusters were found to be highly expressed in the LNP (mRNAgVHSV) fish suggesting there is a difference in the selection pressures between both nucleic acid vaccines and the attenuated VHSV vaccine, with the latter resulting in the selection of more highly probable clones. Interestingly, certain private responses in the LNP (mRNAgVHSV) vaccinated fish also led to significantly higher sum of *pgen* values, with clusters 75229 and 71976 being significantly increased in comparison to DNAgVHSV with cluster 75229 also being significantly higher than PBS fish, indicating that these expansions can contribute to the generation of highly probable clones. Overall, these findings suggest that expanded clusters, particularly after mRNA vaccination, are associated to heterogeneous *pgen* values.

**Fig. 8:**
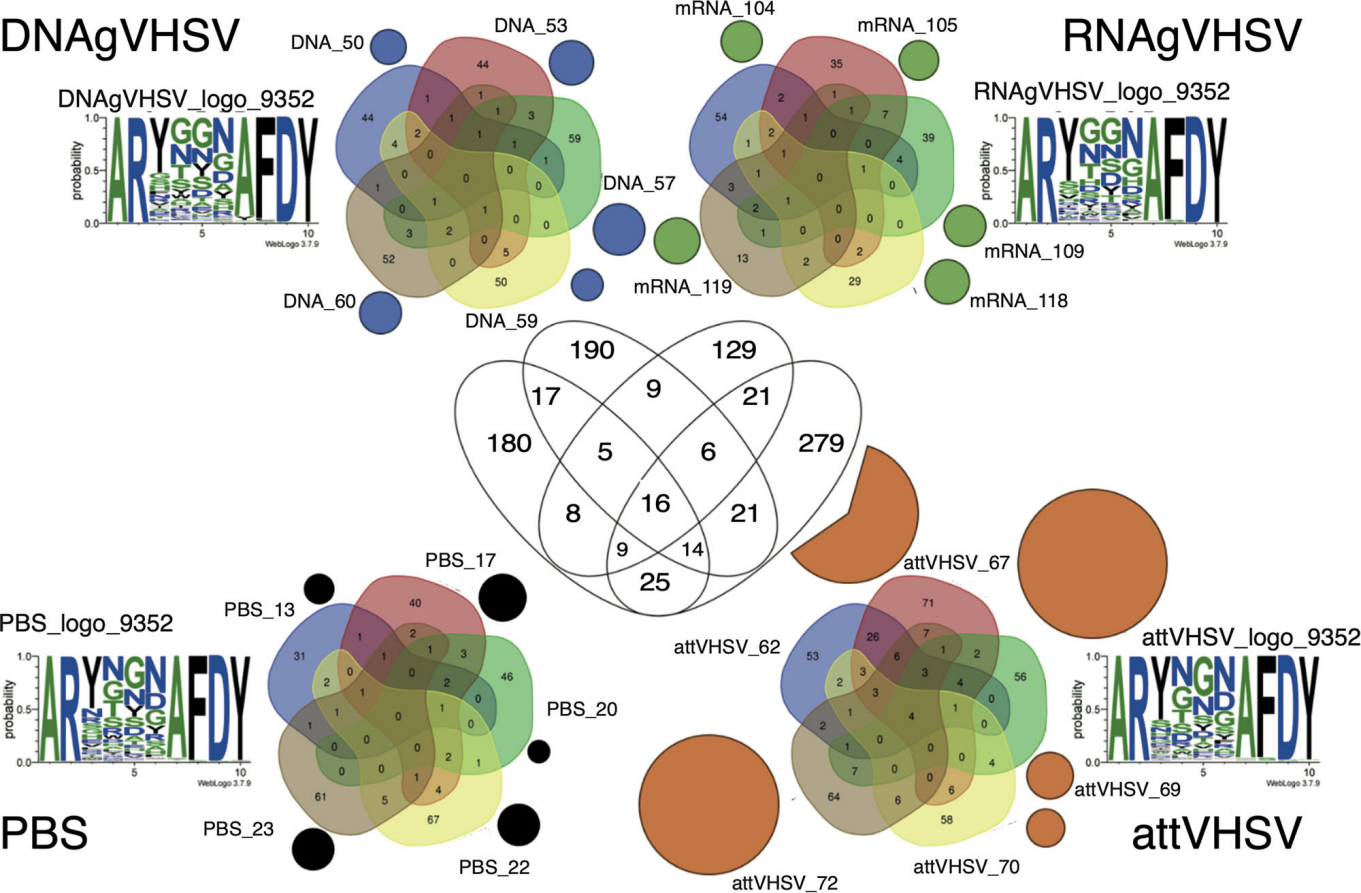
Schematic diagram of cluster 9352 (IGHV1-18*01 F_IGHJ3,5D) relating to the public response. Outer Venn’s depict sharing of clonotypes found in cluster 9352 between fish within groups (subsampling of 20,000 MIDs), whilst the size of the circle represents the sum of expression of clonotypes within cluster 9352 for each fish (subsampling of 100,000 MIDs). Amino acid logos are given for each group. The central Venn depicts sharing of clonotypes with cluster 9352 between groups.

**Fig. 9:**
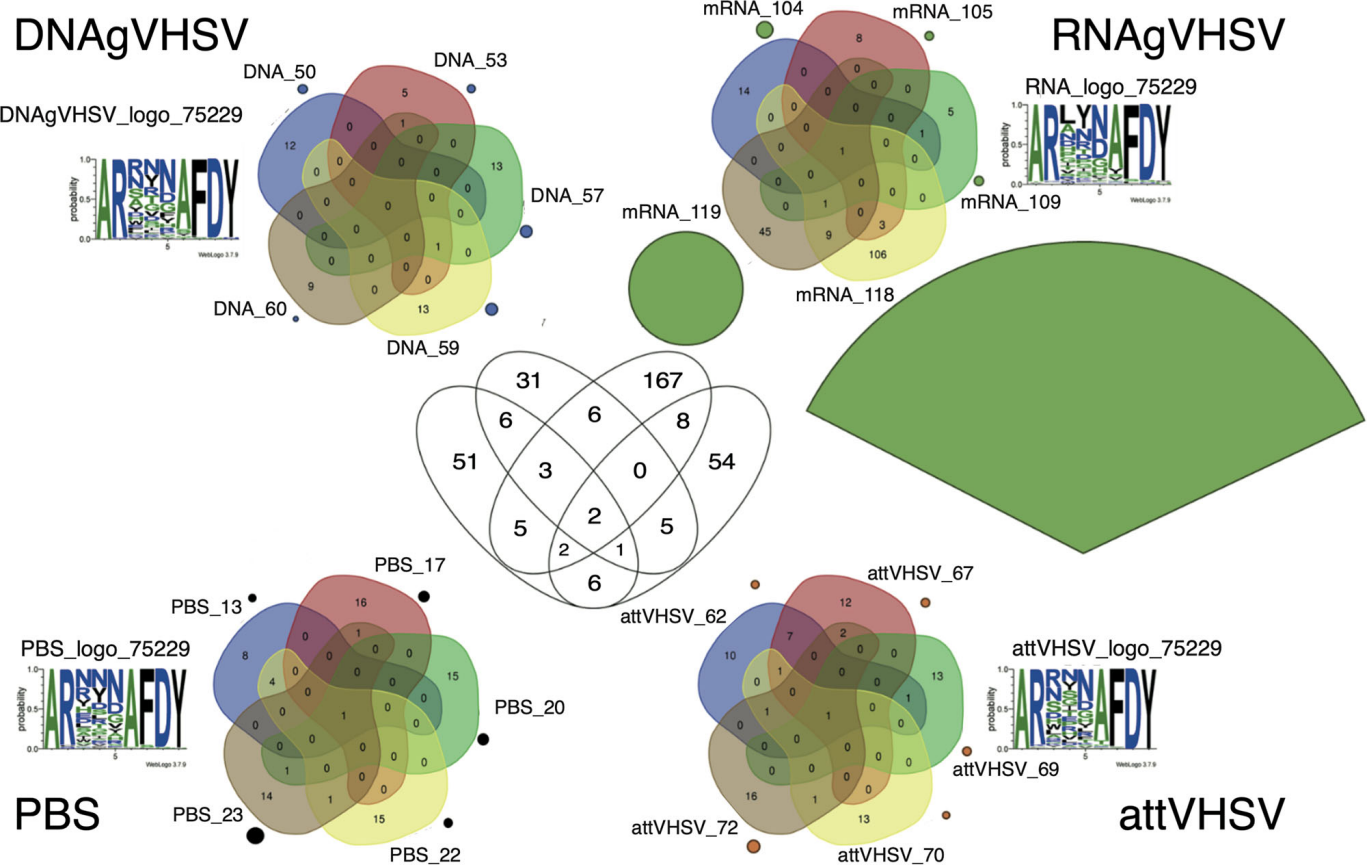
Schematic diagram of cluster 75229 (IGHV6-4*02 F_IGHJ3,5D). Outer Venn’s depict sharing of clonotypes found in cluster 75229 between fish within groups (subsampling of 20,000 MIDs), whilst the size of the circle represents the sum of expression of clonotypes within cluster 75229 for each fish (subsampling of 100,000 MIDs). Amino acid logos are given for each group. The central Venn depicts sharing of clonotypes with cluster 75229 between groups.

To explore the biological significance of these clusters, we selected Clusters 9352 and 75229 for detailed analysis, as they represented key components of the public response driven by AttVHSV and a large private response induced by RNAgVHSV, respectively. Figure 8 illustrates the sharing of clonotypes within the public response Cluster 9352, demonstrating a marked increase in shared clonotypes and expression in AttVHSV-vaccinated fish compared to other groups. Interestingly, DNAgVHSV and RNAgVHSV-vaccinated fish exhibited slight amino acid sequence variations at position 4, where a glycine (G) replaced asparagine (N). Similarly, Fig. 9 presents the sharing of clonotypes within the private response Cluster 75229, showing that while similar sequences were detected at low expression levels across all groups, the RNAgVHSV vaccine drove a distinct response. This was particularly evident in RNAgVHSV_118 and RNAgVHSV_119, both of which exhibited markedly higher expression levels and unique amino acid sequence features, further supporting the hypothesis of a differential immune response induced by the mRNA vaccine in some, but not all, individuals.

## Discussion

In this study, we compared rainbow trout B cell response to three different anti-VHSV vaccines with the same viral antigen leading to the production of neutralising Abs. All three vaccines have previously been shown to induce complete or quasi complete protection against the viral challenge by injection^19,20,22,23^. Deep sequencing of the spleen IgHμ repertoire revealed that these vaccines induce remarkably divergent B cell responses. This comparative approach offers insight into the benefits and limitations of different vaccine platforms, which could help to improve vaccine efficacy and application against viral pathogens.

### The protection induced by mRNA, DNA and attenuated vaccines is associated to different levels of Abs responses

Both DNA and mRNA vaccination drove the production of neutralising antibodies against VHSV, with both showing similar levels of neutralisation. Neutralisation activity of the serum of fish vaccinated with each of the three vaccines was abolished by incubation with the anti-trout IgM monoclonal Ab 1.14, confirming that neutralisation was Ab-dependent regardless of vaccine type^24,27^. The overall titers of anti-VHSV Abs in the serum determined by ELISA were even more contrasted, with high titers in fish vaccinated with the attenuated virus, and very low after immunisation with nucleic acid vaccines. However, all three vaccines afforded full protection^19,20,22,23^. These observations were in line with the idea that neutralising Abs ensure host protection against rhabdoviruses even at low titers^19,28,29^. Similarly in humans, plasma samples from convalescent COVID19 patients did not contain high levels of neutralising activity, but all contained rare but reoccurring Abs specific to the receptor-binding domain which were strongly antiviral^31^. In fact, analysis of the trout IgHμ repertoire showed that the responses induced by the three vaccines did not differ only in intensity.

### mRNA, DNA and attenuated vaccines each have a unique impact on the spleen IgM repertoire

Whole spleen tissue was used for repertoire analysis to ensure we captured the full diversity of IgM expressing B cells present in the spleen. This allowed us to obtain a comprehensive snapshot of the in-situ B cell repertoire at three months post-vaccination, reflecting the full immunological landscape of the spleen at this time point. However, it would be interesting for future studies to test the repertoire of isolated leukocytes, or single cell repertoire analysis to identify if these methods can offer a more targeted and sensitive assessment of immune-related transcripts, thereby enhancing the resolution of clonotype detection.

The limited sharing of top-expressed clonotypes and the overall low levels of shared clonotypes among fish vaccinated with nucleic acid vaccines suggested that these vaccines drove mainly private, though effective, B cell responses. This variability implies that nucleic acid vaccine may stimulate a number of distinct antigen-specific responses directed against distinct epitopes, in contrast to the more homogeneous responses observed with attenuated VHSV, in which public components represent a dominant fraction^26^. We looked for responses shared within and across groups, at different scales (for clonotypes at the whole IgHμ repertoire, VH subgroup, and VH gene levels, and for clusters produced by ATrieGC clustering). Besides the *IGHV1-18* public response to the attenuated vaccine, we found very limited overlap between individuals, for example for IGVH9 within the mRNA vaccinated group. There was evidence of very limited clonotype sharing between groups, particularly for the *IGHV1-18, IGHV6-4, IGHV9-25*, and *IGHV11-25* genes, between the attenuated VHSV and nucleic acid vaccine groups. However, clonotype frequencies of the *IGHV1-18* public response to the attenuated vaccine were at best modest in fish vaccinated with nucleic vaccines. The other common responses were even less important. Importantly, clustering analysis shows that the private responses induced, particularly within the RNA vaccinated fish, were not strictly restricted to a single fish with clusters being found and shared amongst most individuals. Highly expressed clusters (where MIDS were >2000) were mostly expressed at high levels within a single group, and never prominently co-amplified in fish immunised with different vaccines, as illustrated for the two clusters analysed in detail. The top 25 clusters from each group showed a high level of sharing with clusters found in the PBS group but showed there were clusters unique to each vaccination group, particularly the RNA vaccinated fish.

Interestingly, the different vaccines induced very different levels of repertoire perturbation. We found a notable expansion of the top-expressed clonotypes in some fish vaccinated with the LNP (RNAgVHSV) vaccine. Specifically, clonotypes with large compositions (>100 MID counts) were significantly expanded in the LNP (RNAgVHSV) vaccinated group, while no such expansion was observed in the DNA or attenuated VHSV vaccine groups. This was also reflected by the high number of highly expressed clusters in the LNP (RNAgVHSV) vaccinated group. It is of note that the DNAgVHSV vaccine did not provoke a similar dynamic of the B cell repertoire while it led to full protection and neutralising Abs production. When DNA vaccinated fish received a boost at either 1- or 2-months post prime the structure of their repertoire showed no clear increase of clonotypic or cluster expansion compared to PBS. mRNA vaccines are known to elicit strong innate immune responses in humans^36^, driving extensive B cell activation and clonal expansion in response to the expressed antigen. They appear to have the same effect on the fish B cell repertoire. Interestingly, vaccination with LNP (RNAgVHSV) leads to a significant decrease in clonotype diversity within a large subsampling compared to the attenuated VHSV suggesting that clonal expansions induced by the LNP (RNAgVHSV) vaccine may be narrower. Components of the LNP vector, particularly PEGylated lipids, are known to trigger innate immune responses in mammals^37,38^ and likely in fish^21^, which may also contribute to the observed B cell activation in fish and indirectly to repertoire perturbation.

Investigating the known anti-VSHV public clonotypes^24–26^, which encode the IgH of neutralising Abs^27^, shows us that these are somewhat expanded in some individuals 3 months post mRNA and DNA vaccination. This result suggests that they may participate in the protective response demonstrated by both nucleic acid vaccines; however, they did not list among the Top50 most frequent clonotypes in contrast to fish vaccinated with attenuated VHSV, suggesting there may be differences in selection pressures that result in the production of these antibodies.

Overall, our data reveal that each of the three vaccine induces a unique type of B cell response in the trout spleen: mRNA vaccine induces many large expansions in some individuals, convergent to some extent between individuals, thus deeply modifying the structure of the IgHμ repertoire. This is in contrast to the response induced in humans by the mRNA-based BNT162b2 COVID-19 vaccine which elicited a narrow, spike protein-specific antibody response compared to the broader anti-spike response generated by natural infection^33^. These expansions were associated to a modest increase in anti-VHSV IgM titers, and to limited, but protecting neutralising Abs production. The DNA vaccine did not induce large changes of the IgHμ repertoire, with very few large clonotypic expansions and no top frequent expanded cluster. Most large expansions seem to be limited to individual fish. As with the DNA vaccine, the attenuated vaccine production did not induce a large perturbation of the B cell repertoire structure, but led to much higher anti-VHSV and neutralising Ab titers. Public clonotypes and shared clusters were among the top expansions, as described in our previous work^24–26^. Importantly, some of the public clonotypes amplified by the attenuated vaccine were also found expanded, to a limited extent, in most other vaccinated fish, although the difference of frequency between these groups and the control group was not statistically significant.

### Why B cell responses to the three vaccines are so different and why do nucleic acid vaccines not induce the same public expansions observed in attenuated vaccines?

Several non-exclusive explanations may be considered to explain the remarkable divergence between the B cell responses induced by mRNA, DNA and attenuated vaccines.

A first idea is that each vaccine strategy presents a different set of epitopes to the host immune system. Importantly, all fish used in this study belong to the same isogenic, doubled haploid trout line B57^39^, meaning they are homozygous at all loci and share identical MHC class I and class II haplotypes, as well as the same immunoglobulin (and TCR) gene sets. Furthermore, the VHSV G sequence used for our DNA vaccine^19^ and mRNA vaccine^22^ was cloned from the attenuated VHSV strain 25-111 that was used here as vaccine. While viral variants may be produced within the quasi species during infection, the exact same sequence was used across all three immunisation protocols. However, differences in protein folding or presentation in each context could influence epitope availability. Given that the public response encodes neutralising Abs^27^, the fact that all three vaccines provided full protection and induced neutralising antibodies implies that they may not be targeting the same epitopes. This is further illustrated by the sequence logos in nucleic acid vaccinated fish showing variation within the cluster 9352 (public response). These differences need to be explored in more detail to determine if there is an impact on the selection or rearrangement of B cell clones.

Whilst our analysis focused on the IgH (IgM heavy chain repertoire), it is important to note that the functional specificity and diversity of antibodies are also shaped by the pairing of heavy and light chains. Investigating the corresponding immunoglobulin light chain (IgL) could provide further insights into how different vaccine platforms influence B cell repertoire. Future single cell repertoire sequencing will address this question, opening the possibility of in vitro expression and characterisation of relevant antibodies. It may also enhance the sensitivity of the detection of relevant clonal B cell expansions.

Since we analysed IgHμ repertoire in the spleen, another plausible explanation for the absence of B cell expansions is a fast egress of these cells to relocate into other territories. The head kidney is generally considered as the main long term survival for plasma cells in rainbow trout^8^, but this is still debated^40^. We have previously reported that public B cells were found in both the spleen and head kidney after vaccination with attenuated VHSV although these tissues should be considered as distinct compartments^26^. Other potential long term survival niches for plasma cells might be in other recently identified lymphoid tissues^41–43^ or in the peritoneal cavity^44^. Future experiments should evaluate the B cell response to different vaccines in the head kidney to gain additional insights into the longevity, maturation state, and compartmentalisation of vaccine-induced responses. Migrations of B lymphocytes during infections and responses are still poorly understood in salmonids, and will have to be further studied in future works^45^.

Finally, differences in both intensity and clonal composition are likely influenced by the mechanisms of initiation of B cell response, T cell help and selection. We have recently identified Melano-macrophage associated lymphoid aggregates (MLAs) in salmonids as functional counterparts of mammalian germinal centers^6^. MLA formation is induced by infection and immunization with adjuvant, but remains to be characterised after DNA or mRNA vaccination. The inflammatory and costimulatory context after infection with the attenuated virus is clearly different from nucleic acid vaccination. The live attenuated virus can replicate in host cells in many tissues, especially in endothelial cells and should lead to high levels of Ag uptake during the infectious process. In contrast, nucleic acid vaccines typically produce relatively short-term transient antigens after mRNA^22,46^ or DNA^19,20^ delivery, typically within non-APCs. This might lead to potentially limited exposure and different patterns of antigen uptake and presentation. How and where such differences might favour private expansions of B cells expressing low *pgen* rearrangements versus public responses involving typically high *pgen* clonotypes, remains to be understood. The difference of intensity and composition of responses induced by mRNA and DNA vaccination with an Ag encoded by the same sequence in fish sharing the same genetic background is remarkable and underline the deep divergence between the responses they induce. Of note, it has been previously observed that rainbow trout TCRβ repertoire modification induced by DNA vaccination against VHSV were minimal compared to viral infection^47^.

While such direct comparisons between vaccination strategies remain largely unexplored in mammalian models, our findings warrant the need for further investigation into the fundamental differences in immune response dynamics to different vaccines in mammals. The observed intrinsic divergence in B cell responses despite identical antigen sequences suggests that nucleic acid vaccines may influence antigen processing and epitope presentation in ways distinct from live-attenuated vaccines. This parallels questions in human vaccinology, where mRNA and DNA vaccines elicit differing antibody responses, potentially impacting neutralising epitope targeting and long-term immunity. However, further experiments are needed in fish to establish the difference in persistence of long-term memory between the different vaccines. Furthermore, the possibility of rapid B cell egress from the spleen raises broader considerations regarding tissue-specific immune responses, a key factor in optimising vaccine efficacy across vertebrates. Whilst this study used an unmodified mRNA vaccine, human vaccine development has already shifted to using modified mRNA platforms, a topic requiring further study in the fish model. Understanding these mechanisms could contribute to the refinement of both fish and human vaccines, particularly in the development of nucleic acid-based platforms for long-lasting and protective immunity.

## Methods

### Experimental design

Adult rainbow trout were reared, vaccinated, boosted and infected at the fish facilities at the Institute National de la Recherche en Agriculture et environnement (INRAE, Jouy-en-Josas, France). Fish were PIT tagged 21 days before vaccination to allow for individual monitoring and kept at 15 °C. A first experiment using juvenile rainbow trout (350 g) (*N* = 12 per group) (2-year-old) were vaccinated using a DNA vaccine, other controls can be seen in Fig. S7. A dose of 10 µg of pcDNA3.1 plasmid encoding for the VHSV glycoprotein or encoding for eGFP was used per fish. 2 year old fish were used due to the efficacy of the DNA vaccine being proven previously^19^. All fish were vaccinated by intramuscular injection (IM) at day 0, followed by a booster injection at day 60. The 60-day time point was used for the booster DNA vaccination to provide the best possible complement to a previous experiment which showed a minimal public response at 90 days in the blood of rainbow trout after a booster vaccination at 30 days (as explained in Supplementary Text).”

A second experiment using juvenile rainbow trout (100 g) (1-year-old) were vaccinated using an mRNA vaccine (*N* = 12 per group), other controls can be seen in Fig. S7. As before all fish were injected by IM route with 5 μg of LNPs encapsulating an mRNA encoding for the VHSV glycoprotein or encoding for eGFP was used per fish at day 0, followed by a booster injection at day 36. At day 90 in both experiments, fish were culled (overdose of Tricaine followed by destruction of the brain) with serum and spleen samples being taken. All relevant groups were kept in the same tank within each experiment, ensuring that all fish were subjected to the same conditions. An independent experiment was carried out for the DNAgVHSV vaccine with the methodology being shown in the Supplementary Text.

Young adult fish were used for these experiments to characterise the modifications of the B cell repertoire induced by DNA and mRNA vaccines, in comparison to the response to the attenuated vaccine we previously characterised^25,27^. We are aware that fish have to be vaccinated at an earlier developmental stage in commercial aquaculture.

All experiments were carried out in accordance with the European Union guidelines for the handling and welfare of laboratory animals (https://ec.europa.eu/environment/chemicals/lab_animals/index_en.htm). The experimental protocols were approved by the INRAE institutional ethics committee “COMETHEA” (DAP 18-37).

### Vaccine production

The attenuated vaccine is comprised of a thermoresistant VHSV Strain 25.111 that does not replicate well at 16 ^o^C^23^. The same VHSV glycoprotein gene was used to produce mRNA for LNP formulation, and to construction the DNA vaccine vector in pcDNA3.1^19^. The LNP (RNAgVHSV) vaccine was prepared as previously described in Ayad et al.^22^.

### ELISA and neutralisation assays against VHSV

Total anti-VHSV IgM antibodies in serum samples were quantified using an indirect enzyme-linked immunosorbent assay (ELISA). Ninety-six-well plates (Nunc MaxiSorp, Thermo Fisher Scientific, USA) were coated overnight at room temperature with 100 μL per well of a whole-virus lysate of VHSV strain 07.71, diluted 1:1000 in Dulbecco’s phosphate-buffered saline (DPBS; Life Technologies, USA). The amount of virus introduced in each well corresponded to a VHSV suspension containing 2 × 10⁵ pfu. The virus had been propagated in epithelioma papulosum cyprini (EPC) cells, purified by ultracentrifugation through a glycerol cushion, and inactivated using β-propiolactone. Following coating, plates were blocked for 1 h at 37 °C with 200 μL per well of DPBS containing 10% milk powder (UGAP, France). Plates were washed three times with 400 μL per well of PBS containing 0.05% Tween-20 (PBS-T; Euromedex, France) using an automatic plate washer (Thermo Scientific, USA). Sera were serially diluted (1:100 to 1:12,800) in DPBS supplemented with 1% bovine serum albumin (BSA; Euromedex, France), and 100 μL of each dilution was added to the wells. Plates were incubated for 1 h at 37 °C and subsequently washed three times with PBST. Mouse monoclonal anti-trout IgM (0.5 μg/mL in DPBS with 1% BSA) was then added (100 μL per well) and incubated for 1 h at 37 °C. After washing, plates were incubated for 1 h at 37 °C with 100 μL per well of horseradish peroxidase (HRP)-conjugated goat anti-mouse IgG (Southern Biotech, USA), diluted to 0.1 μg/mL in DPBS with 1% BSA. Plates were washed again with PBS-T before adding 100 μL per well of TMB substrate (1-Step Ultra TMB ELISA, Thermo Fisher Scientific, USA). After 30 min of incubation at room temperature in the dark, the reaction was stopped by adding 100 μL per well of 0.5 M sulfuric acid (VWR, France). Absorbance was measured at 450 nm with reference at 620 nm using a microplate reader (Multiskan FC, Thermo Fisher Scientific, France).

Serum neutralisation assays were conducted following the method described by Castro et al. (2022). To assess the role of IgM, the anti-IgM monoclonal antibody 1.14 was used as outlined in Castro et al. (2013). Briefly, 5 μg of 1.14 Ab was pre-incubated with serum for 3 h at room temperature before being incorporated into the virus neutralisation assay, with the rest of the protocol remaining unchanged.

### Library preparation for B-cell repertoire

Libraries were prepared for those samples which demonstrated evidence of a protective response (DNA, mRNA and attenuated vaccine) alongside PBS injected controls. Libraries were synthesized following the methodology outlined in Castro et al.^26^. Total RNA was extracted from one half of the spleen in 1 ml of TRI-reagent (Sigma Aldrich) following the manufacturer’s instructions. Using full spleen tissue allowed to get a comprehensive snapshot of the in-situ B cell repertoire at three months post-vaccination, reflecting the full immunological landscape of the spleen at this time point. The RNA was then washed using 80% ethanol and dissolved in RNase-free water and stored at −80 °C until further use. RNA concentration was determined by nanodrop (Sigma Aldrich). One microgram of total RNA from the spleen served as the initial starting material for library construction. All primers for this study can be found in Supplementary Table S1. Reverse transcription was carried out using the SMARTer 5′ RACE kit (Clontech) as per the manufacturer’s guidelines, starting at the C region-specific primers for IgM (Cmu2) and incorporates the SMARTer II A oligonucleotide (universal adaptor) at the 5′ end. Second strand synthesis was carried out using the SeqAmp DNA polymerase (Clontech), Cmu2 primer and Rd2p_UTD_5′Race primer (Contains an illumina adaptor and a 15 random nucleotide sequence (Unique Molecular Identifier (UMI))) under the following thermal cycling conditions: 98 °C for 3 min, 59 °C for 4 min, 72 °C for 10 min, 4 °C hold. The resulting double-stranded cDNA (ds-cDNA) was purified using Mag-Bind total pure next generation sequencing beads (VWR) at a ratio of 1:1.2 (sample:beads) following the manufacturer’s instructions. The cDNA library was further amplified in a two-stage PCR. The ds-cDNA was amplified with DreamTaq (Thermofisher) using 0.3 μM forward primers (containing Illumina adaptor sequences and six fish barcode nucleotides (FBD) (Rd2_FBD_Rd2p) and the reverse primer for the C-region Cmu1 under the following thermal cycling conditions: 95 °C for 5 mins, 28 cycles (95 °C for 30 s, 59 °C for 35 s, 72 °C for 60 s), 72 °C for 7 min, 4 °C hold. The resulting PCR product was purified by gel purification using the NucleoSpin gel and PCR clean-up kit (Clontech). The second stage of the PCR was carried out using the same forward primer and a reverse primer containing an Illumina adaptor, an 8-nucleotide barcode (FBT) and 2 random nucleotides at the 5′ end for different samples (Rd1_2N_FBT_Cmu1). Cycling was as follows 95 °C for 5 min, 15 times (95 °C for 30 s, 64 °C for 35 s, 72 °C for 60 s), 72 °C for 7 min, 4 °C hold. The second PCR product was purified using the Mag-Bind Total RNA pure NGS beads with a ratio of 1:1.6 (sample:beads) to remove small fragments. A small aliquot of the purified samples was run on a 1% agarose gel to ensure amplification of the correct size (~700 bp) had taken place.

### B-cell repertoire sequencing and analysis

Libraries were sequenced using the NextSeq 2000 Machine in paired end runs (2 × 300-bp) (P1 - 600 cycles kit) at the Institute for Integrative Biology of the Cell (I2BC), Gif-sur-Yvette. Initial data analysis involved demultiplexing of the samples using bcl-convert 4.1.5. Adaptor trimming was performed using Cutadapt 3.2 and quality control of the reads was assessed using FastQC v0.11.5. Reads were filtered and merged to create consensus sequences using pRESTO. Consensus sequences were then annotated using IMGT/HighV-Quest using the arlee genome according to IMGT gene tables and standardised IMGT nomenclature of IgH genes. Resulting in each consensus sequence containing a unique identifier (UID), IGHV gene, IGHJ gene, a Cμ1 gene and complementarity determining region 3 (CDR3) region. The UID and CDR3 regions were combined to create a molecular identifier (MID) facilitating subsequent sample identification and quantification. The expression of each clonotype, defined by the V region, J region and CDR3, could then be calculated by identifying the corresponding number of MID barcodes.

A diversiTR web-based interface was used to aid in the extraction of the data. To select top 50 clonotypes or to identify shared clonotypes the expression of clonotypes across all vaccination conditions was calculated using the MID subsamples and used to rank or identify clonotypes initially at a 20,000 subsampling. Since we observed very low sharing of top50 clonotypes in DNAgVHSV and LNP (RNAgVHSV) vaccinated groups, we increased the subsampling size to 100,000 clonotypes, in order to increase the chance of detecting clonotypes of interest. To visualize the public response to VHSV across all groups a dotplot was generated using ggplot2 (V3.3.4) in R (v4.4.0). The AnalyzAIRR package (https://github.com/i3-unit/AnalyzAIRR) was used to calculate various measures of clonotype diversity; Gini index, and Shannon diversity index^48^.

### Probability of generation

Probability of generation (*Pgen*) was calculated for selected clonotypes using the OLGA model^49^ with V/J restrictions. The software uses an IGoR model trained with unproductive sequences of BCRs of rainbow trouts^50^. The probabilities for each clonotype were calculated based on the nucleotide sequence along with the V and J gene(s) and CDR3 sequence. For clonotypes with ambiguous J gene, selection of the J gene was based on the position of the V gene.

### Clustering of clonotypes

Clustering of similar clonotypes was performed using ATrieGC (https://github.com/statbiophys/ATrieGC), a software that quickly performs a single-linkage clustering. A clonotype is put in a cluster if the cluster contains at least one clonotype with a CDR3 having a Hamming distance smaller or equal to 1 (in amino acid space). Note that clonotypes with different CDR3 lengths will never be in the same cluster. Sequence logos for each cluster were generated using WebLogo 3^51^, providing a graphical representation of conserved sequence motifs and amino acid distributions within each cluster.

### Statistical analysis

Statistical analysis was carried out using Graphpad Prism 9 for one- and two-way ANOVAs. R (v4.4.3) was used to carry out one- and two-sided Kolmogorov-Smirnov tests.

## Supplementary information

Supplementary Information

## Data Availability

Sequence data that support the findings of this study have been deposited in the BioProject National Center for Biotechnology Information database under accession number PRJNA1245658.Here is the link to the bioproject: https://dataview.ncbi.nlm.nih.gov/object/PRJNA1245658.

## References

[1] Burnet, F. M. A modification of jerne’s theory of antibody production using the concept of clonal selection. *CA: A Cancer J. Clin.* **26**, 119–121 (1976). 10.3322/canjclin.26.2.119 816431

[2] Hirano, M., Das, S., Guo, P. & Cooper, M. D. The Evolution of Adaptive Immunity in Vertebrates. in *Advances in Immunology* (ed. Alt, F. W.)109, 125–157 (Academic Press, 2011). 10.1016/B978-0-12-387664-5.00004-2 21569914

[3] Flajnik, M. F. A cold-blooded view of adaptive immunity. *Nat. Rev. Immunol.* **18**, 438–453 (2018). 29556016 10.1038/s41577-018-0003-9 PMC6084782

[4] Parra, D., Takizawa, F. & Sunyer, J. O. Evolution of B cell immunity. *Annu. Rev. Anim. Biosci.* **1**, 65–97 (2013). 25340015 10.1146/annurev-animal-031412-103651 PMC4203447

[5] Bjørgen, H. & Koppang, E. O. Anatomy of teleost fish immune structures and organs. in *Principles of Fish Immunology: From Cells and Molecules to Host Protection* (eds. Buchmann, K. & Secombes, C. J.) 1–30. 10.1007/978-3-030-85420-1_1 (Springer International Publishing, Cham, 2022).

[6] Shibasaki, Y. et al. Cold-blooded vertebrates evolved organized germinal center–like structures. *Sci. Immunol.* **8**, eadf1627 (2023). 37910630 10.1126/sciimmunol.adf1627 PMC11152321

[7] Waly, D., Muthupandian, A., Fan, C.-W., Anzinger, H. & Magor, B. G. Immunoglobulin VDJ repertoires reveal hallmarks of germinal centers in unique cell clusters isolated from zebrafish (Danio rerio) lymphoid tissues. *Front. Immunol*. **13**, 1058877 (2022). 10.3389/fimmu.2022.1058877 PMC9772432 36569890

[8] Bromage, E. S., Kaattari, I. M., Zwollo, P. & Kaattari, S. L. Plasmablast and Plasma cell production and distribution in trout immune tissues1. *J. Immunol.* **173**, 7317–7323 (2004). 15585855 10.4049/jimmunol.173.12.7317

[9] Zwollo, P., Mott, K. & Barr, M. Comparative analyses of B cell populations in trout kidney and mouse bone marrow; establishing “B cell signatures”. *Dev. Comp. Immunol.* **34**, 1291–1299 (2010). 20705088 10.1016/j.dci.2010.08.003 PMC2945407

[10] Heesters, B. A., van der Poel, C. E., Das, A. & Carroll, M. C. Antigen presentation to B cells. *Trends Immunol.* **37**, 844–854 (2016). 27793570 10.1016/j.it.2016.10.003

[11] Fillatreau, S. et al. The astonishing diversity of Ig classes and B cell repertoires in teleost fish. *Front. Immunol.* **4**, 28 (2013). 23408183 10.3389/fimmu.2013.00028 PMC3570791

[12] Zhang, Y.-A. et al. IgT, a primitive immunoglobulin class specialized in mucosal immunity. *Nat. Immunol.* **11**, 827–835 (2010). 20676094 10.1038/ni.1913 PMC3459821

[13] Cossarini-Dunier, M. Protection against enteric redmouth disease in rainbow trout, Salmo gairdneri Richardson, after vaccination with Yersinia ruckeri bacterin. *J. Fish. Dis.* **9**, 27–33 (1986).

[14] Rijkers, G., Frederix-Wolters, E. M. H. & Muiswinkel, W. V. The immune system of Cyprinid fish. The effect of antigen dose and route of administration on the development of immunological memory in carp (Cyprinus carpio). in *Phylogeny of Immunological Memory* (Elsevier/North-Holand, 1980).

[15] Rijkers, G. T., Frederix-Wolters, E. M. & van Muiswinkel, W. B. The immune system of cyprinid fish. Kinetics and temperature dependence of antibody-producing cells in carp (Cyprinus carpio). *Immunology* **41**, 91–97 (1980). 7000695 PMC1458243

[16] Scharsack, J. P. & Franke, F. Temperature effects on teleost immunity in the light of climate change. *J. Fish. Biol.* **101**, 780–796 (2022). 35833710 10.1111/jfb.15163

[17] Collins, C., Lorenzen, N. & Collet, B. DNA vaccination for finfish aquaculture. *Fish. Shellfish Immunol.* **85**, 106–125 (2019). 30017931 10.1016/j.fsi.2018.07.012

[18] Ma, J., Bruce, T. J., Jones, E. M. & Cain, K. D. A review of fish vaccine development strategies: conventional methods and modern biotechnological approaches. *Microorganisms* **7**, 569 (2019). 31744151 10.3390/microorganisms7110569 PMC6920890

[19] Boudinot, P., Blanco, M., de Kinkelin, P. & Benmansour, A. Combined DNA immunization with the glycoprotein gene of viral hemorrhagic septicemia virus and infectious hematopoietic necrosis virus induces double-specific protective immunity and nonspecific response in rainbow trout. *Virology* **249**, 297–306 (1998). 9791021 10.1006/viro.1998.9322

[20] Lorenzen, N. et al. Protective immunity to VHS in rainbow trout (Oncorhynchus mykiss, Walbaum) following DNA vaccination. *Fish. Shellfish Immunol.* **8**, 261–270 (1998). 10.1016/j.fsi.2004.10.009 15722229

[21] Dahl, L. O. S. et al. Implementation of mRNA–Lipid Nanoparticle Technology in Atlantic Salmon (Salmo salar). *Vaccines* **12**, 788 (2024). 39066426 10.3390/vaccines12070788 PMC11281423

[22] Ayad, C. et al. An LNP-mRNA vaccine protects fish against rhabdovirus infection. *Vaccine* **53**, 126957 (2025). 40031086 10.1016/j.vaccine.2025.126957

[23] De Kinkelin, P., Bearzotti-Le Berre, M. & Bernard, J. Viral hemorrhagic septicemia of rainbow trout: selection of a thermoresistant virus variant and comparison of polypeptide synthesis with the wild-type virus strain. *J. Virol.* **36**, 652–658 (1980). 6780698 10.1128/jvi.36.3.652-658.1980 PMC353692

[24] Castro, R. et al. Teleost Fish mount complex clonal IgM and IgT responses in spleen upon systemic viral infection. *PLOS Pathog.* **9**, e1003098 (2013). 23326228 10.1371/journal.ppat.1003098 PMC3542120

[25] Magadan, S. et al. Origin of public memory b cell clones in fish after antiviral vaccination. *Front. Immunol*. **9**, 2115 (2018). 10.3389/fimmu.2018.02115 PMC6170628 30319606

[26] Castro, R. et al. Clonotypic IgH response against systemic viral infection in pronephros and spleen of a teleost fish. *J. Immunol.* **208**, 2573–2582 (2022). 35577368 10.4049/jimmunol.2200088

[27] Castro, R. et al. Cutting Edge: neutralizing public antibody responses are an ancient form of defense conserved in fish and mammals. *J. Immunol.* **207**, 371–375 (2021). 34233911 10.4049/jimmunol.2100149 PMC11152318

[28] Both, L. et al. Passive immunity in the prevention of rabies. *Lancet Infect. Dis.* **12**, 397–407 (2012). 22541629 10.1016/S1473-3099(11)70340-1

[29] Pattanaik, A. & Mani, R. S. WHO’s new rabies recommendations: implications for high incidence countries. *Curr. Opin. Infect. Dis.* **32**, 401 (2019). 31305491 10.1097/QCO.0000000000000578

[30] Muecksch, F. et al. Increased memory B cell potency and breadth after a SARS-CoV-2 mRNA boost. *Nature* **607**, 128–134 (2022). 35447027 10.1038/s41586-022-04778-y PMC9259484

[31] Robbiani, D. F. et al. Convergent antibody responses to SARS-CoV-2 in convalescent individuals. *Nature* **584**, 437–442 (2020). 32555388 10.1038/s41586-020-2456-9 PMC7442695

[32] He, B. et al. Comparative global B cell receptor repertoire difference induced by SARS-CoV-2 infection or vaccination via single-cell V(D)J sequencing. *Emerg. Microbes Infect.* **11**, 2007–2020 (2022). 35899581 10.1080/22221751.2022.2105261 PMC9377262

[33] Kotagiri, P. et al. B cell receptor repertoire kinetics after SARS-CoV-2 infection and vaccination. *Cell Rep.* **38**, 110393 (2022). 35143756 10.1016/j.celrep.2022.110393 PMC8801326

[34] Deluca, D., Wilson, M. & Warr, G. W. Lymphocyte heterogeneity in the trout, Salmo gairdneri, defined with monoclonal antibodies to IgM. *Eur. J. Immunol.* **13**, 546–551 (1983). 6347695 10.1002/eji.1830130706

[35] Spisak, N., Athènes, G., Dupic, T., Mora, T. & Walczak, A. M. Combining mutation and recombination statistics to infer clonal families in antibody repertoires. *eLife* **13**, e86181 (2024). 39120133 10.7554/eLife.86181 PMC11441979

[36] Verbeke, R., Hogan, M. J., Loré, K. & Pardi, N. Innate immune mechanisms of mRNA vaccines. *Immunity* **55**, 1993–2005 (2022). 36351374 10.1016/j.immuni.2022.10.014 PMC9641982

[37] Alameh, M.-G. et al. Lipid nanoparticles enhance the efficacy of mRNA and protein subunit vaccines by inducing robust T follicular helper cell and humoral responses. *Immunity* **54**, 2877–2892.e7 (2021). 34852217 10.1016/j.immuni.2021.11.001 PMC8566475

[38] Li, C. et al. Mechanisms of innate and adaptive immunity to the Pfizer-BioNTech BNT162b2 vaccine. *Nat. Immunol.* **23**, 543–555 (2022). 35288714 10.1038/s41590-022-01163-9 PMC8989677

[39] Quillet, E., Dorson, M., Le Guillou, S., Benmansour, A. & Boudinot, P. Wide range of susceptibility to rhabdoviruses in homozygous clones of rainbow trout. *Fish. Shellfish Immunol.* **22**, 510–519 (2007). 17085058 10.1016/j.fsi.2006.07.002

[40] Jenberie, S., van der Wal, Y. A., Jensen, I. & Jørgensen, J. B. There and back again? A B cell’s tale on responses and spatial distribution in teleosts. *Fish & Shellfish Immunol.* 109479, 10.1016/j.fsi.2024.109479 (2024). 10.1016/j.fsi.2024.109479 38467322

[41] Løken, O. M., Bjørgen, H., Hordvik, I. & Koppang, E. O. A teleost structural analogue to the avian bursa of Fabricius. *J. Anat.* **236**, 798–808 (2020). 31877586 10.1111/joa.13147 PMC7163591

[42] Resseguier, J. et al. Identification of a pharyngeal mucosal lymphoid organ in zebrafish and other teleosts: Tonsils in fish?. *Sci. Adv.* **9**, eadj0101 (2023). 37910624 10.1126/sciadv.adj0101 PMC10619939

[43] Tacchi, L. et al. Nasal immunity is an ancient arm of the mucosal immune system of vertebrates. *Nat. Commun.* **5**, 5205 (2014). 25335508 10.1038/ncomms6205 PMC4321879

[44] Jenberie, S. et al. Virus-specific antibody secreting cells reside in the peritoneal cavity and systemic immune sites of Atlantic salmon (Salmo salar) challenged intraperitoneally with salmonid alphavirus. *Dev. Comp. Immunol.* **157**, 105193 (2024). 38729458 10.1016/j.dci.2024.105193

[45] Jiménez-Guerrero, R. et al. Differentiation and traffic of IgM+ B cells between focal dark spots in skeletal muscle of Atlantic salmon, lymphoid and adipose tissues. *Fish. Shellfish Immunol.* **139**, 108858 (2023). 37302676 10.1016/j.fsi.2023.108858

[46] Qin, F. et al. A guide to nucleic acid vaccines in the prevention and treatment of infectious diseases and cancers: from basic principles to current applications. *Front. Cell Dev. Biol.* **9**, 633776 (2021). 34113610 10.3389/fcell.2021.633776 PMC8185206

[47] Boudinot, P. et al. The glycoprotein of a fish rhabdovirus profiles the virus-specific T-cell repertoire in rainbow trout. *J. Gen. Virol.* **85**, 3099–3108 (2004). 15448373 10.1099/vir.0.80135-0

[48] Mhanna, V. et al. AnalyzAIRR: A user-friendly guided workflow for AIRR data analysis. *ImmunoInformatics* **19**, 100052 (2025). 10.1016/j.immuno.2025.100052 PMC7619189 42305137

[49] Sethna, Z., Elhanati, Y., Callan, C. G. Jr, Walczak, A. M. & Mora, T. OLGA: fast computation of generation probabilities of B- and T-cell receptor amino acid sequences and motifs. *Bioinformatics* **35**, 2974–2981 (2019). 30657870 10.1093/bioinformatics/btz035 PMC6735909

[50] Marcou, Q., Mora, T. & Walczak, A. M. High-throughput immune repertoire analysis with IGoR. *Nat. Commun.* **9**, 561 (2018). 29422654 10.1038/s41467-018-02832-w PMC5805751

[51] Crooks, G. E., Hon, G., Chandonia, J.-M. & Brenner, S. E. WebLogo: a sequence logo generator. *Genome Res.* **14**, 1188–1190 (2004). 15173120 10.1101/gr.849004 PMC419797

