# Supplementary material for: Divergent B-cell repertoire remodelling by mRNA, DNA and live attenuated vaccines in fish
Source: NPJ Vaccines. 2025 Jul 24;10:166. doi: 10.1038/s41541-025-01232-8 (PMC12289986; doi:10.1038/s41541-025-01232-8)
Supplement: Supplementary file 1 — Supplementary Information [file 41541_2025_1232_MOESM1_ESM.pdf]

Supplementary Materials for

Divergent B-cell repertoire remodelling by mRNA, DNA and live attenuated vaccines in fish

Dean Porter[1], Catherine Collins[1,2,3], Andrea Mazzolini[4], Luc Jouneau[1, 5], Vanessa Mhanna[6], Céline Coiffier[7], Mathilde Peruzzi[1], Yan Jaszczyszyn[8], Encarnita Mariotti-Ferrandiz[6], Francois Huetz[9], Bertrand Collet[1], Thierry Mora[4], Aleksandra M Walczak[4], Bernard Verrier[7,10] and Pierre Boudinot[1, *]

[1] Université Paris-Saclay, INRAE, UVSQ, VIM, 78350 Jouy-en-Josas, France
[2] School of Biological, Earth and Environmental Sciences, University College Cork, T23 TK30, Cork, Ireland
[3] Environmental Research Institute, University College Cork, T23 XE10, Cork, Ireland
[4] Laboratoire de physique de l'école normale supérieure (PSL University), CNRS, Sorbonne Université and Université Paris Cité, 75005 Paris, France.
[5] Université de Paris-Saclay, INRAE, BREED, 78350 Jouy-en-Josas, France
[6] Sorbonne Université, INSERM, Immunology-Immunopathology-Immunotherapy (i3), F-75005 Paris, France.
[7] UMR 5305: Laboratoire de Biologie Tissulaire et d'Ingénierie Thérapeutique, 69007, Lyon, France
[8] Université Paris-Saclay, CEA, CNRS, Institute for Integrative Biology of the Cell (I2BC), 91198 Gif-sur-Yvette Cedex, France
[9] Institut Pasteur, Université Paris Cité, INSERM UMR1222, Antibodies in Therapy and Pathology, 75015 Paris, France.
[10] Adjuvatis, Batiment Laennec 60, Avenue Rockefeller 69008 Lyon, France
* Corresponding Author – pierre.boudinot@inrae.fr

Supplementary text: Independent DNA vaccination experiment.

In an independent vaccination trial with DNAgVHSV (DNA) and PBS (Ctr) for controls, blood IgHm repertoire was sequenced from immunized fish of the same isogenic line (B57) after a prime boost protocol. The table below shows the MID counts for all 8 public clonotypes in each replicate with the average frequency calculated from a 20,000 MID subsampling. The detailed description of the immunization protocol, library contruction and Miseq Illumina sequencing are given below in the section "Detailed methodology".

Counts and frequency of anti-VHSV public clonotypes in peripheral blood leucocytes

Clonotype	F101_Ctr_Bl_d59	F102_Ctr_Bl_d59	F103_Ctr_Bl_d59	F107_Ctr_Bl_d59	F067_DNA_Bl_d59	F068_DNA_Bl_d59	F069_DNA_Bl_d59	F070_DNA_Bl_d59
ARYNGDAFDY_IGHV1-18*02 F_IGHJ2D,3,5D,6	0	0	0	1	0	1	0	0
ARYNNDAFDY_IGHV1-18*02 F_IGHJ2D,3,5D,6	0	0	0	0	0	0	0	0
ARYNNNAFDY_IGHV1-18*02 F_IGHJ2D,3,5D,6	0	0	0	0	0	8	0	0
ARYDNNAFDY_IGHV1-18*02 F_IGHJ2D,3,5D,6	16	2	0	0	0	1	0	1
ARYTGNAFDY_IGHV1-18*02 F_IGHJ2D,3,5D,6	0	0	0	0	0	0	0	0
ARYSGDAFDY_IGHV1-18*02 F_IGHJ2D,3,5D,6	0	0	0	0	0	0	0	0
ARYGGNAFDY_IGHV1-18*02 F_IGHJ2D,3,5D,6	1	0	0	1	0	0	8	0
ARYGDNAFDY_IGHV1-18*02 F_IGHJ2D,3,5D,6	1	0	0	0	0	0	0	1
Frequency	0.000275				0.00025			

Detailed methodology

Vaccine

The same DNA vaccine was used in both experiments with PBS injected fish acting as a control for both experiments.

Experimental design

Rainbow trout (B57), 10 months old and with an average weight of 26 g, were obtained from Pisciculture Expérimentale facility, INRAE. The fish were maintained in freshwater at 16 °C. The fish were pit tagged into the abdominal cavity and left to recover for 21 days prior to administration of trial vaccines to allow for individual monitoring. Fish were vaccinated at day 0 by intramuscular injection with either 1 x PBS or vaccinated with DNAgVHSV (10 µg per fish), and boosted at day 30 with the same dose. At day 59, fish were anaesthetized with MS222 (Sigma Aldrich) and 50 µL of blood was extracted per fish with a 1 ml syringe and 29 G needle (BD Micro-Fine). Blood was extracted from the caudal vein, with needle inserted ventrally. Whole blood from each individual was added directly to tube containing 600 µL RLT buffer (RNAeasy, Minikit, Qiagen) and ceramic beads (1.4 mm, Qiagen).

Preparation of Illumina MiSeq CDR3 IgHµ repertoire libraries

RNA extraction

Six µL of β-mercaptoethanol (Sigma Aldrich) was added to the 50 µL blood sample in RTL buffer + ceramic beads, and the sample was homogenized as for spleen above, spun at 13000 rpm for 3 minutes and 325 µL transferred to an extraction column (RNAeasy kit, Qiagen) and processed

followings manufacturer's instructions. RNA concentrations were obtained using a Nanodrop 2000 (ThermoFisher Scientific).

cDNA synthesis and purification of products

First strand cDNA synthesis was performed following manufacturer's instructions using the SMARTer RACE 5'/3' kit (Takara Bio, Clontech) and the Cmu2 reverse primer. The primers used in this study can be found in *(20)*. The Cmu2 primer binds to the Cμ2 domain of the CDR3, which results in excluding cDNA synthesis from IgHδ mRNA which contains the Cμ1 domain. 1 μg RNA was added to each reaction. The final reaction was not diluted.Second strand cDNA synthesis was performed using the Rd2p_UID_5'RACE primer (Integrated DNA Technologies). This is a primer consisting of an Illumina adapter sequence, a UID (Unique Molecular Identifier) region of 15 nucleotides randomly generated which gives up to 10^6 different combinations (*45*), and the complementary sequence to the Smarter II A oligo added to 5' end of 1st round cDNA synthesized products. The UID portion of the primer will allow for detection of PCR bias in subsequent amplification of the cDNA, and clonotype frequencies. The reaction mix was as follows: SeqAmp PCR Buffer 1 x (Takara, Clontech), Rd2p_UID_5'RACE primer (1.5 μM), Seq Amp DNA polymerase 0.6 μL, and water to a final volume of 20μL. 10 μL of first strand cDNA synthesis reaction was added to the 20 μL second strand reaction and incubated at 98°C @ 3 min., 59°C @ 4 min., 72°C @ 10 min. The reaction was purified using Mag-Bind Total (RNA) pure NGS (Omega Biotek) at a 1:1 ratio of beads to sample and following manufacturer's instructions.

PCR amplification and purification of products

First round amplification on cDNA products was performed using the primer Cmu1 which binds internally to the primer Cmu2 on the Cμ2 domain, and a RD2_FBDn_Rd2p primer which incorporates a universal adapter for Illumina sequencing and a unique index of 6 nucleotides (TruSeq, Illumina) used to identify individual samples when pooled for sequencing. Each reaction mix consisted of 1 x DreamTaq Hot Start PCR mix (ThermoFisher Scientific), 1.5 μM each primer, 5.0 μL purified cDNA and water to a final volume of 50 μL. Cycling conditions were 95 °C @ 5 min. x 1, 95 °C @ 30 sec., 59 °C @ 35 sec., 72 °C @ 60 sec. x 28, 72 °C @ 7 min x 1. Amplification products were run on a 1 % TAE gel, excised and purified according to manufacturer's instructions using the Nucleospin Gel and PCR Clean-up kit (Macherey-Nagel).

Second round PCR was performed to add the universal adapter for Illumina sequencing to the 3' end of the first round PCR product, using the primer RD2_FBDn_Rd2p as previously and primer Rd1_2N_Cμ1. The primer Rd1_2N_Cμ1 (Table 2) consists of the Illumina adapter and Cmu1 primer sequence which has been truncated by 4 nucleotides at its 5' end with 4 nucleotides added at its 3' end. The reaction was as follows: 1 x DreamTaq Hot Start PCR mix, 1.5 μM each primer, 2.0 μL purified first round product and water to a final volume of 50 μL. Cycling conditions were 95 °C @ 5 min. x 1, 95 °C @ 30 sec., 64 °C @ 35 sec., 72 °C @ 60 sec. x 15, 72 °C @ 7 min x 1. A 5μL aliquot of second round PCR was run on a 1 % TAE gel to confirm correct amplification and the remainder was purified using the Mag-Bind Total (RNA) pure NGS as previously, using a bead to sample ratio of 1:0.6.

Preparation of samples for MiSeq Sequencing

Concentration of purified PCR products was obtained using a Qubit Fluorometer (ThermoFisher Scientific) and samples diluted to 5 ng/uL DNA for running on a Bioanalyser 2100 (Agilent), using an Agilent DNA 1000 chip, to confirm product size, and to obtain molarity.

Appropriate sample volumes were pooled to give equimolar concentrations of each sample to a final total concentration of 5 nM in 20 µL. Pooled samples underwent a final purification using Mag-Bind Total (RNA) pure NGS as previously with a 1.2: 1 ratio of beads: sample. Sequencing was performed at the CurieCoreTech platform, Institute Curie, Paris, utilising MiSeq 2 x 300 bp paired-end sequencing and the MiSeq Reagent kit v3 (600 cycles) (Illumina). PhiX was added at 30 % to each pool prior to sequencing.

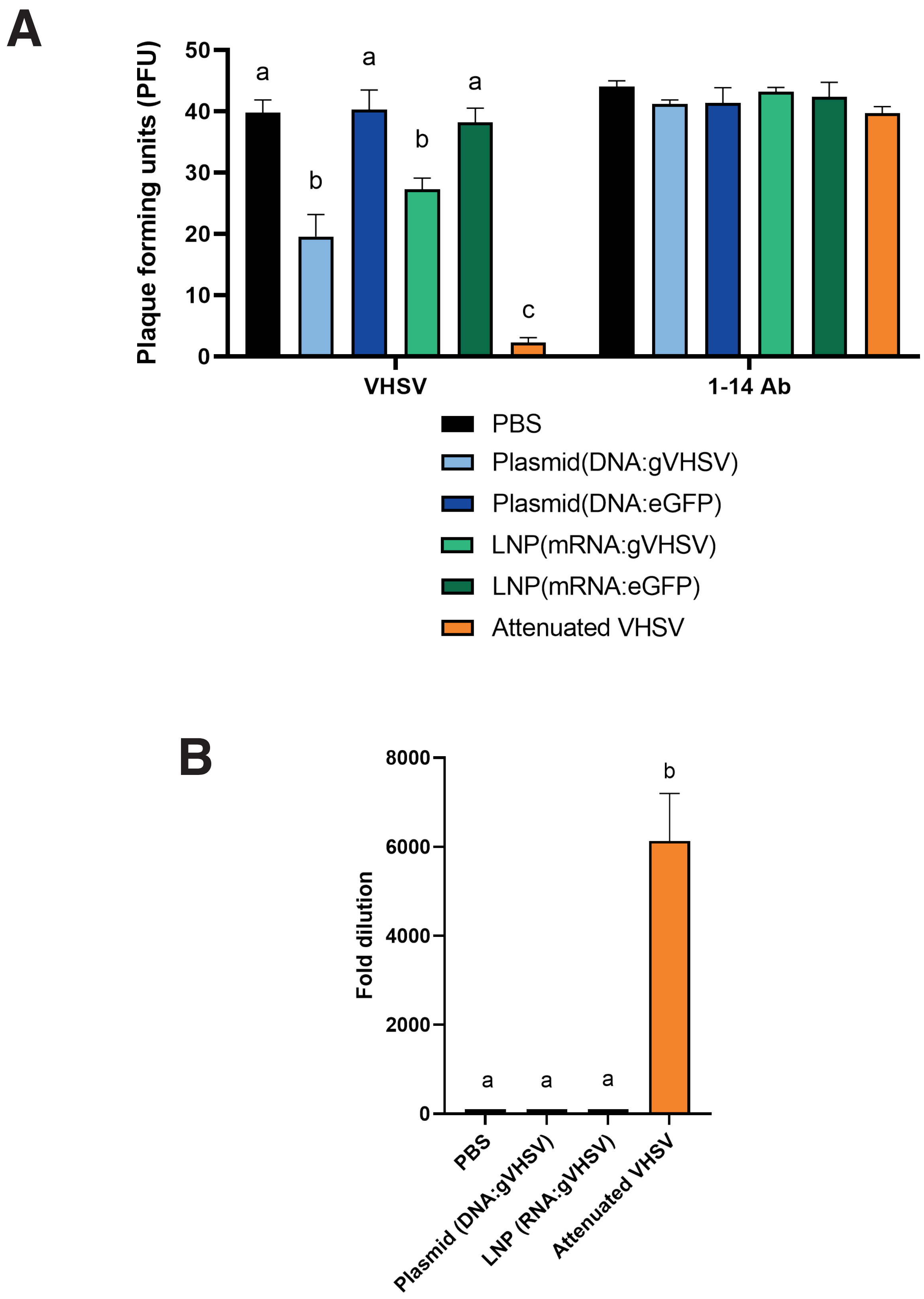

Figure S1 - Correlates of protection between DNA, RNA and attenuated vaccines to VHSV.
A . Serum neutralisation showing levels of protection offered by antibodies in the serum. VHSV neutralization was abolished in presence of the anti-rainbow trout IgM 1.14 monoclonal Ab, confirming that neutralisation was IgM-dependent. a, b and c denote significant differences where $P < 0.05$
B. ELISA showing levels of anti-VHSV antibodies in the serum of fish at three months post-vaccination.

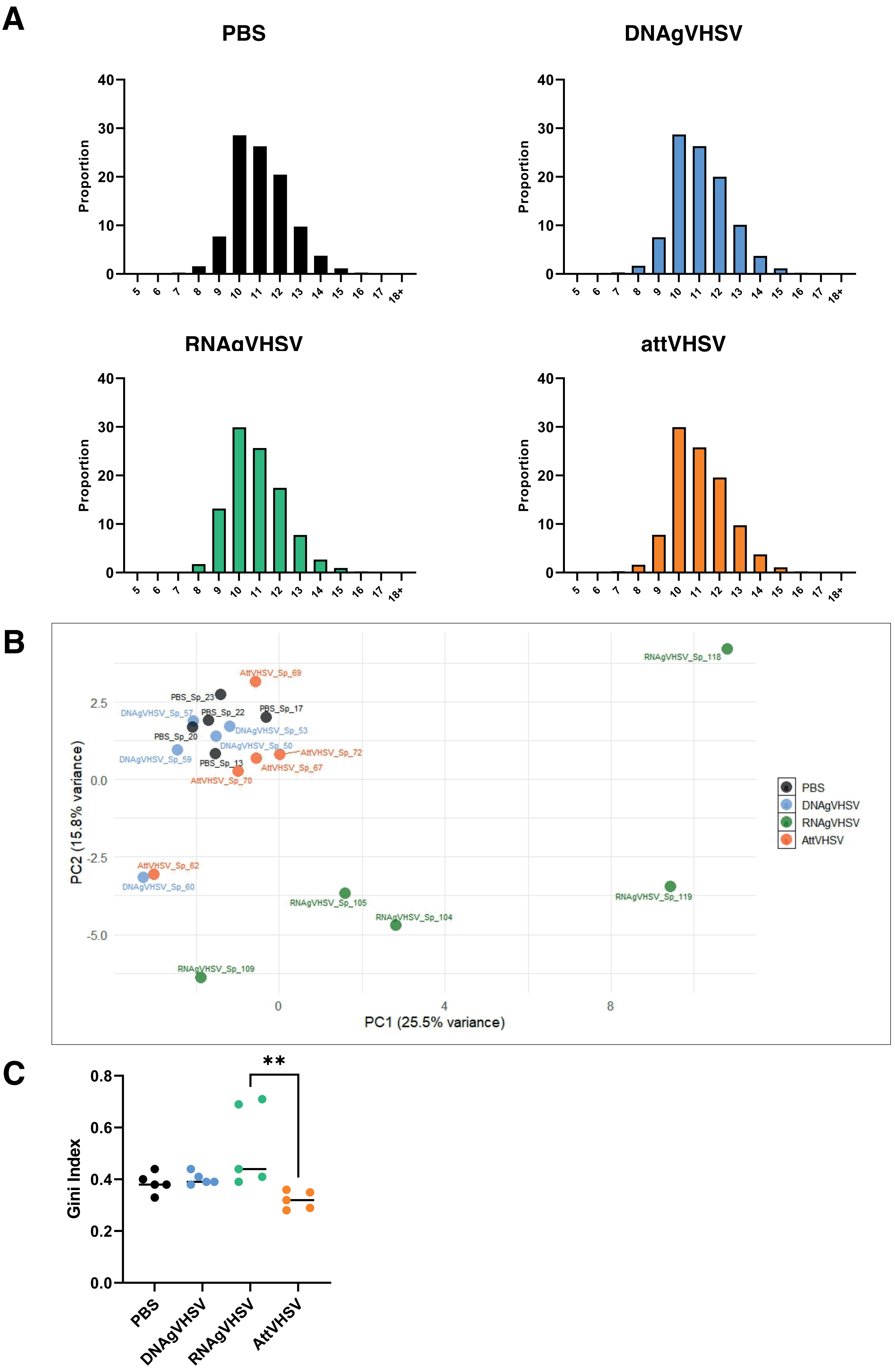

Figure S2 - Characteristics of the clonotype composition three months after vaccination with nucleic acid vaccines. A . CDR3 length distribution for each vaccine type. B Principal Component Analysis (PCA) of IGHV gene usage profiles, showing clustering by vaccine group. C Measures of clonotype diversity between different vaccination groups using Gini indice.

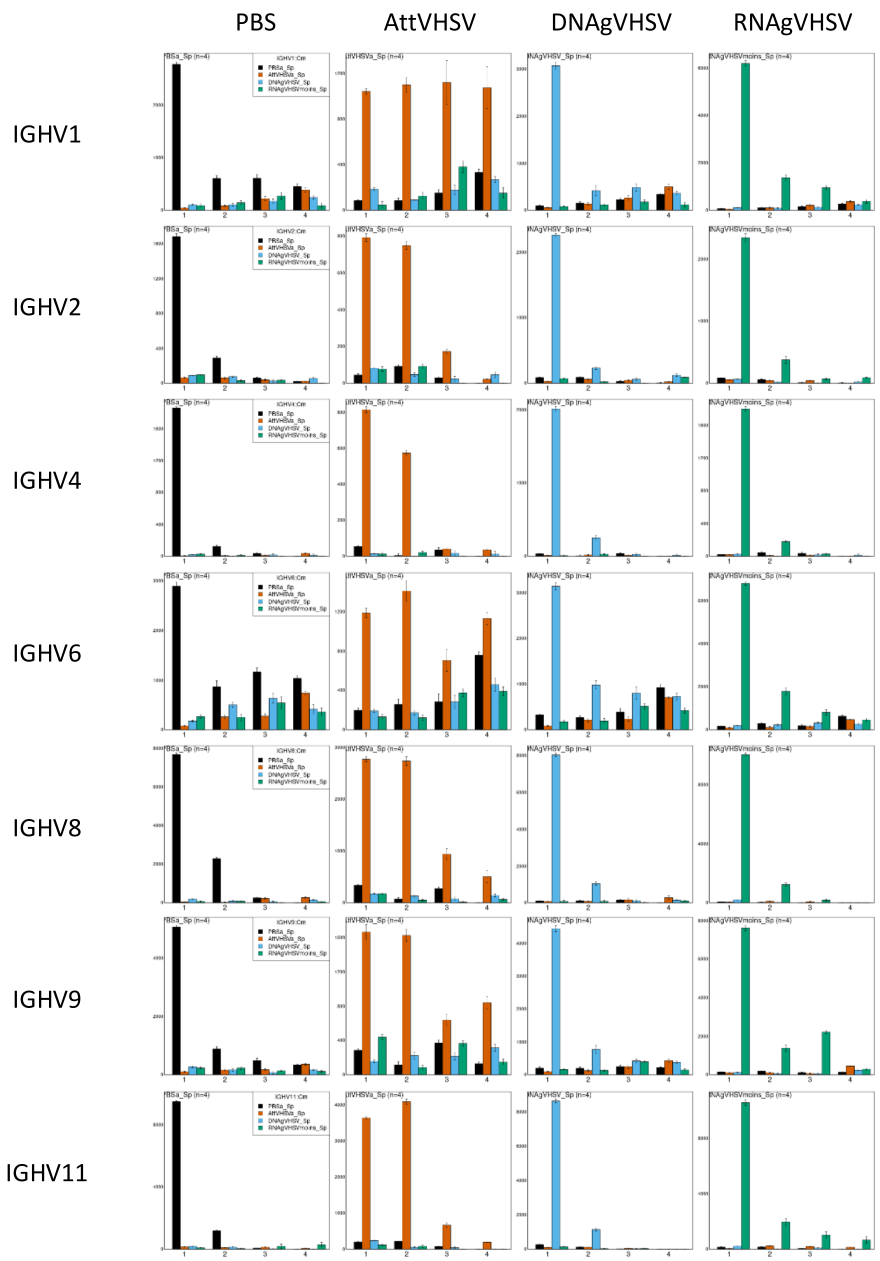

Figure S3 - Highly shared clonotypes within IGHV subgroups. Barplots showing cumulative expression of the top 50 clonotypes shared by n individuals (*n* = 1 fish or 2 or 3 or 4) for a given IGHV subgroup within each vaccinated group after 90 days post initial vaccination. Individual barplot show total expression and sharing of the top 50 clonotypes for that condition compared to other conditions with PBS (in black), AttVHSV (in orange), DNAgVHSV (in blue) and RNAgVHSV (in green). X axis (1, 2, 3 and 4) demonstrates the level of sharing within each condition. Cumulative expression is based on the average MID count of 10 subsamplings of 10,000 per individual.

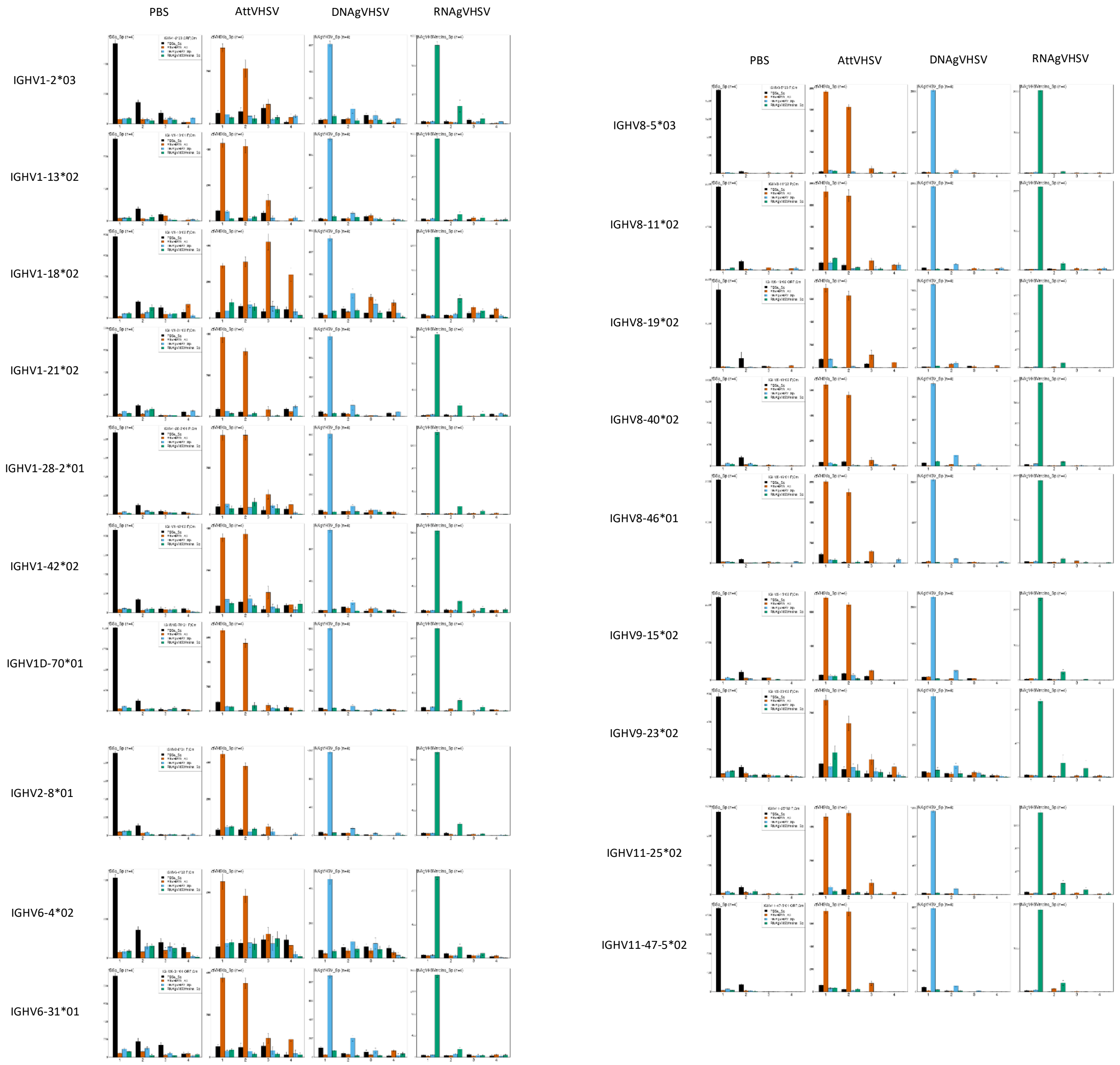

Figure S4 – Highly shared clonotypes at the IGHV gene level. A Barplots showing cumulative expression of the top 50 clonotypes shared by n individuals for a given IGHV (*n* = 1, 2, 3 or 4) within each vaccinated group after 90 days post initial vaccination. Individual barplots show total expression and sharing of the top 50 clonotypes for that condition compared to other conditions with PBS (in black), AttVHSV (in orange), DNAgVHSV (in blue) and RNAgVHSV (in green). X axis (1, 2, 3 and 4) demonstrates the level of sharing within each condition. Cumulative expression is based on the average MID count of 10 subsamplings of 1,000.

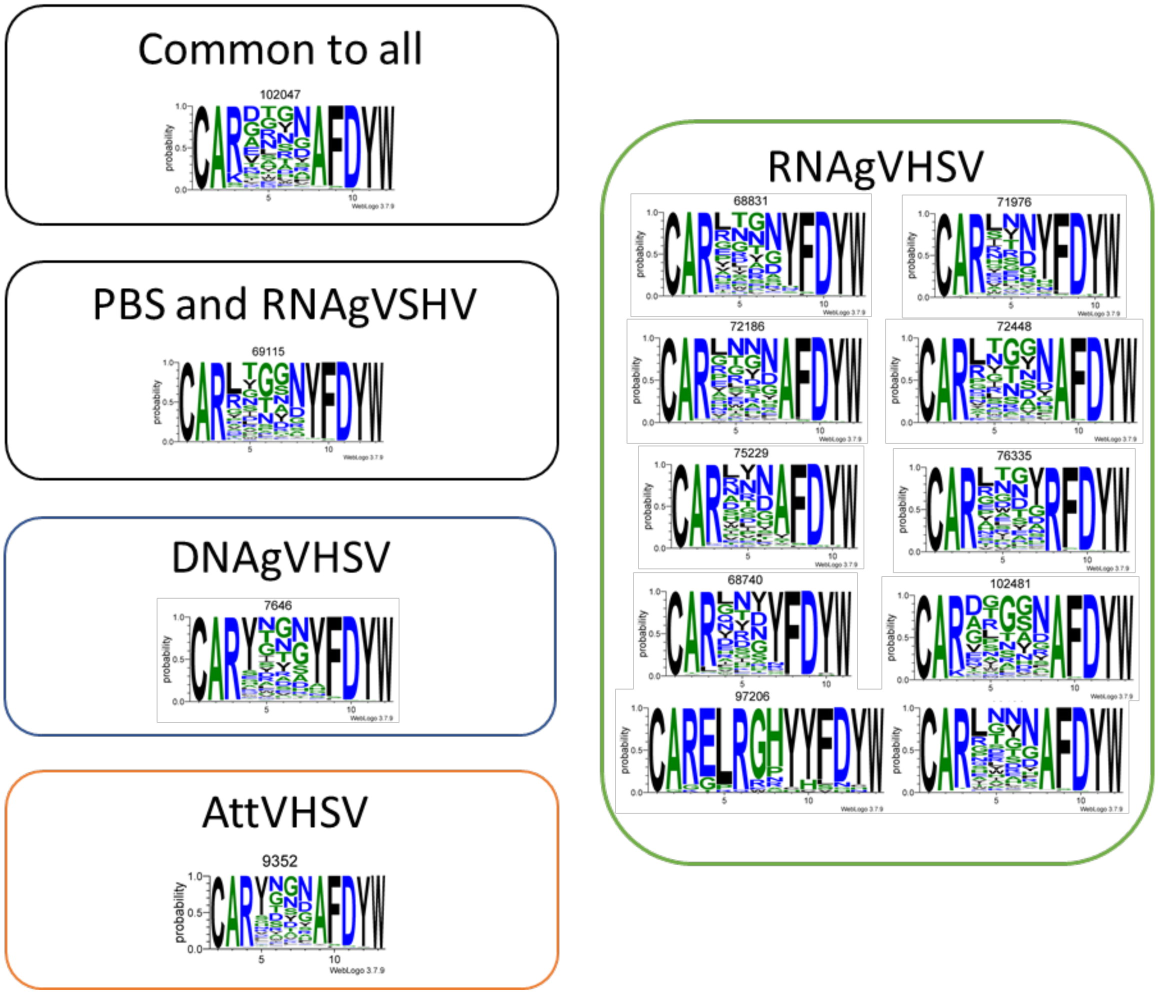

Figure S5 – Amino acid sequence logos for the most expressed clusters shown on Figure 7B. Amino acid sequence logos represent the most highly expressed IgHμ clonotypes across different vaccination groups. Clusters were selected based on having a summed MID count >2000 in at least one fish per group (see Figure 7 B, C and D). Sequences from all fish within each group were used to generate the logos. Sequence logos were generated using WebLogo3 (*47*)

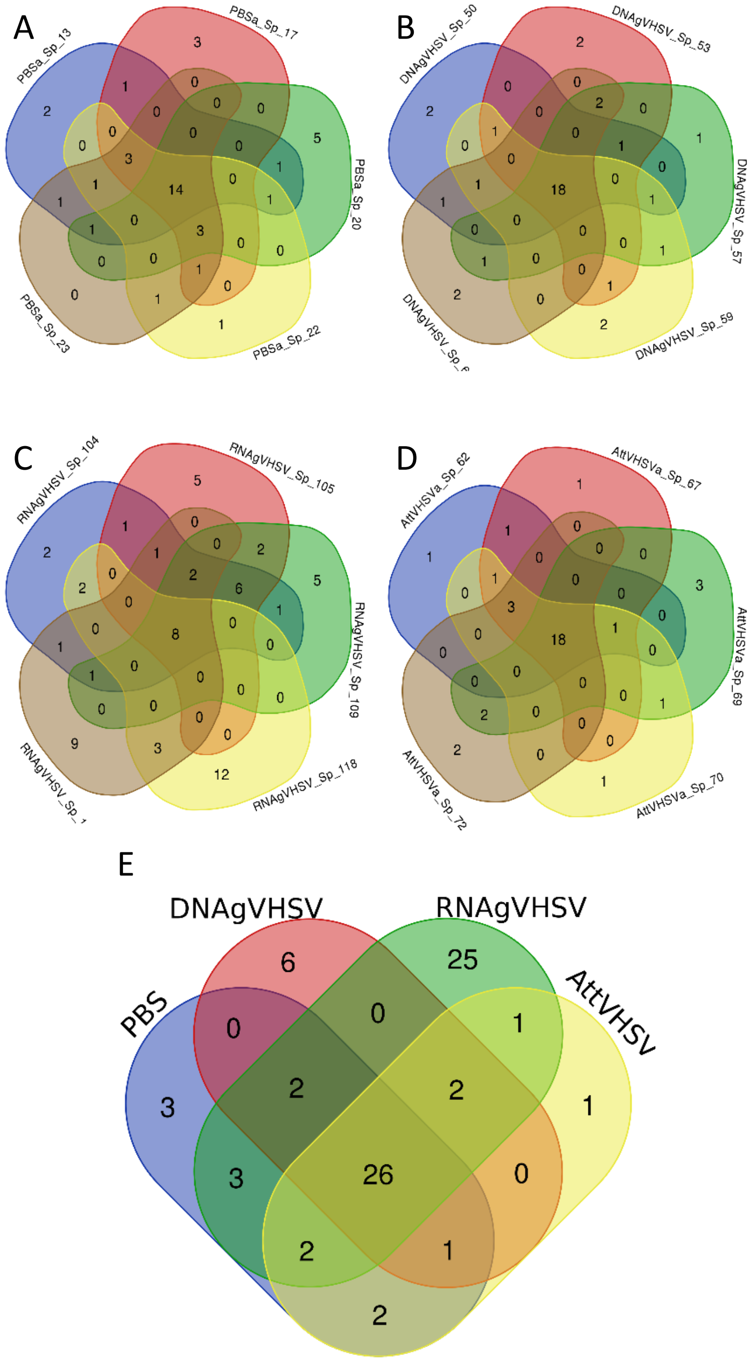

Figure S6 – Sharing of the top 25 clusters in each vaccination group. **A-D** Venn diagrams illustrating the overlap of the top 25 clusters among individual fish within each vaccination group. The clusters identified in each group were used to generate the top cluster list for panel E. **E** Venn diagram comparing the top cluster lists across different vaccination groups.

A

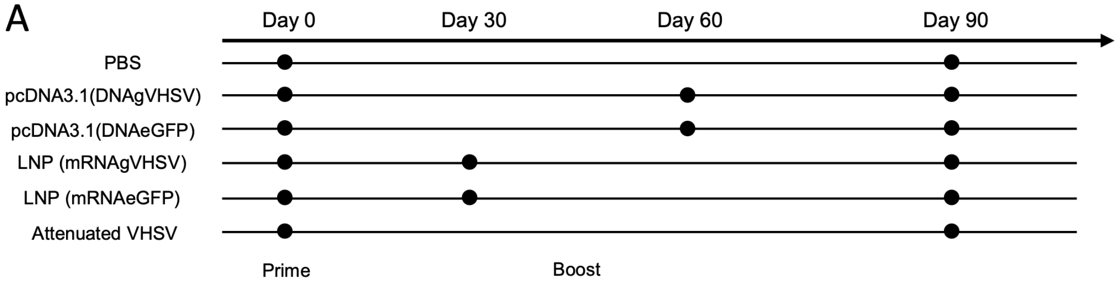

B

Group	Prime	Boost	Amount per fish
PBS	D0	-	100 µL of 1x PBS
pcDNA3.1(DNAgVHSV)	D0	D60	10 µg
pcDNA3.1(DNAeGFP)	D0	D60	10 µg
pcDNA3.1(DNAgVHSV)	D0	D30	5 µg
pcDNA3.1(DNAgVHSV)	D0	D30	5 µg
Attenuated VHSV (Strain 25.111)	D0	-	5000 pfu

Figure S7 – Experimental Design. A Experimental design for experiments assessing nucleic acid vaccines and attenuated VHSV. Sampling was performed on day 90. **B** Table showing vaccine dosages performed using a prime-boost protocol.

Table S1 – Primers used in this study

Primer	Sequence (5’ - 3’)
Rd2p_UMI_5RACE	GTGACTGGAGTTCAGACGTGTGCTCTTCCGATCTNNNNNNNNNNNNNNNAAGCAGTGGTATCAACGCAGAGT
Cmu2	AGAGACGGCTGCTGCAGATATTCC
Cmu1	CACATTGCGCAAGAGGGAACAA
Rd2_FBD1_Rd2p	CAAGCAGAAGACGGCATACGAGATCGTGATGTGACTGGAGTTCAGACGTGTGCTCTTCCGATCT
Rd2_FBD2_Rd2p	CAAGCAGAAGACGGCATACGAGATACATCGGTGACTGGAGTTCAGACGTGTGCTCTTCCGATCT
Rd2_FBD3_Rd2p	CAAGCAGAAGACGGCATACGAGATGCCTAAGTGACTGGAGTTCAGACGTGTGCTCTTCCGATCT
Rd2_FBD4_Rd2p	CAAGCAGAAGACGGCATACGAGATTGGTCAGTGACTGGAGTTCAGACGTGTGCTCTTCCGATCT
Rd2_FBD5_Rd2p	CAAGCAGAAGACGGCATACGAGATCACTGTGTGACTGGAGTTCAGACGTGTGCTCTTCCGATCT
Rd2_FBD6_Rd2p	CAAGCAGAAGACGGCATACGAGATATTGGCGTGACTGGAGTTCAGACGTGTGCTCTTCCGATCT
Rd2_FBD9_Rd2p	CAAGCAGAAGACGGCATACGAGATCTGATCGTGACTGGAGTTCAGACGTGTGCTCTTCCGATCT
Rd2_FBD10_Rd2p	CAAGCAGAAGACGGCATACGAGATAAGCTAGTGACTGGAGTTCAGACGTGTGCTCTTCCGATCT
Rd2_FBD11_Rd2p	CAAGCAGAAGACGGCATACGAGATGTAGCCGTGACTGGAGTTCAGACGTGTGCTCTTCCGATCT
Rd2_FBD12_Rd2p	CAAGCAGAAGACGGCATACGAGATTACAAGGTGACTGGAGTTCAGACGTGTGCTCTTCCGATCT
Rd2_FBD13_Rd2p	CAAGCAGAAGACGGCATACGAGATTTGACTGTGACTGGAGTTCAGACGTGTGCTCTTCCGATCT
Rd2_FBD14_Rd2p	CAAGCAGAAGACGGCATACGAGATGGAACTGTGACTGGAGTTCAGACGTGTGCTCTTCCGATCT
Rd2_FBD15_Rd2p	CAAGCAGAAGACGGCATACGAGATTGACATGTGACTGGAGTTCAGACGTGTGCTCTTCCGATCT
Rd2_FBD16_Rd2p	CAAGCAGAAGACGGCATACGAGATGGACGGGTGACTGGAGTTCAGACGTGTGCTCTTCCGATCT
Rd2_FBD22_Rd2p	CAAGCAGAAGACGGCATACGAGATCGTACGGTGACTGGAGTTCAGACGTGTGCTCTTCCGATCT
Rd2_FBD26_Rd2p	CAAGCAGAAGACGGCATACGAGATGCTCATGTGACTGGAGTTCAGACGTGTGCTCTTCCGATCT
Rd2_FBD31_Rd2p	CAAGCAGAAGACGGCATACGAGATATCGTGGTGACTGGAGTTCAGACGTGTGCTCTTCCGATCT
Rd2_FBD38_Rd2p	CAAGCAGAAGACGGCATACGAGATAGCTAGGTGACTGGAGTTCAGACGTGTGCTCTTCCGATCT
Rd1_2N_FBT1_CMu1	AATGATACGGCGACCACCGAGATCTACACTAGATCGCACACTCTTTCCCTACACGACGCTCTTCCGATCTTTGCGCAAGAGGGAACAAAGTC
Rd1_2N_FBT2_CMu1	AATGATACGGCGACCACCGAGATCTACACCTCTCTATACACTCTTTCCCTACACGACGCTCTTCCGATCTTTGCGCAAGAGGGAACAAAGTC
Rd1_2N_FBT3_CMu1	AATGATACGGCGACCACCGAGATCTACACTATCCTCTACACTCTTTCCCTACACGACGCTCTTCCGATCTTTGCGCAAGAGGGAACAAAGTC
Rd1_2N_FBT4_CMu1	AATGATACGGCGACCACCGAGATCTACACAGAGTAGAACACTCTTTCCCTACACGACGCTCTTCCGATCTTTGCGCAAGAGGGAACAAAGTC
Rd1_2N_FBT5_CMu1	AATGATACGGCGACCACCGAGATCTACACGTAAGGAGACACTCTTTCCCTACACGACGCTCTTCCGATCTTTGCGCAAGAGGGAACAAAGTC
Rd1_2N_FBT6_CMu1	AATGATACGGCGACCACCGAGATCTACACACGCATAACACTCTTTCCCTACACGACGCTCTTCCGATCTTTGCGCAAGAGGGAACAAAGTC
Rd1_2N_FBT7_CMu1	AATGATACGGCGACCACCGAGATCTACACAAGGAGTAACACTCTTTCCCTACACGACGCTCTTCCGATCTTTGCGCAAGAGGGAACAAAGTC

Rd1_2N_FBT8_CMu1	AATGATACGGCGACCACCGAGATCTACACCTAAGCCTACACTCTTTCCCTACACGACGCTCTTCCGATCTTTGCGCAAGAGGGAACAAAGTC
Rd1_2N_FBT10_CMu1	AATGATACGGCGACCACCGAGATCTACACCGTCTAATACACTCTTTCCCTACACGACGCTCTTCCGATCTTTGCGCAAGAGGGAACAAAGTC
Rd1_2N_FBT11_CMu1	AATGATACGGCGACCACCGAGATCTACACTCTCTCCGACACTCTTTCCCTACACGACGCTCTTCCGATCTTTGCGCAAGAGGGAACAAAGTC
Rd1_2N_FBT13_CMu1	AATGATACGGCGACCACCGAGATCTACACTCGACTAGACACTCTTTCCCTACACGACGCTCTTCCGATCTTTGCGCAAGAGGGAACAAAGTC
Rd1_2N_FBT15_CMu1	AATGATACGGCGACCACCGAGATCTACACTTCTAGCTACACTCTTTCCCTACACGACGCTCTTCCGATCTTTGCGCAAGAGGGAACAAAGTC
Rd1_2N_FBT16_CMu1	AATGATACGGCGACCACCGAGATCTACACCCTAGAGTACACTCTTTCCCTACACGACGCTCTTCCGATCTTTGCGCAAGAGGGAACAAAGTC
Rd1_2N_FBT17_CMu1	AATGATACGGCGACCACCGAGATCTACACGCGTAAGAACACTCTTTCCCTACACGACGCTCTTCCGATCTTTGCGCAAGAGGGAACAAAGTC
Rd1_2N_FBT18_CMu1	AATGATACGGCGACCACCGAGATCTACACCTATTAAGACACTCTTTCCCTACACGACGCTCTTCCGATCTTTGCGCAAGAGGGAACAAAGTC
Rd1_2N_FBT20_CMu1	AATGATACGGCGACCACCGAGATCTACACAAGGCTATACACTCTTTCCCTACACGACGCTCTTCCGATCTTTGCGCAAGAGGGAACAAAGTC
Rd1_2N_FBT21_CMu1	AATGATACGGCGACCACCGAGATCTACACGAGCCTTAACACTCTTTCCCTACACGACGCTCTTCCGATCTTTGCGCAAGAGGGAACAAAGTC
Rd1_2N_FBT22_CMu1	AATGATACGGCGACCACCGAGATCTACACTTATGCGAACACTCTTTCCCTACACGACGCTCTTCCGATCTTTGCGCAAGAGGGAACAAAGTC
